# Conformer Generation for Structure-Based Drug Design: How Many and How Good?

**DOI:** 10.1021/acs.jcim.3c01245

**Published:** 2023-10-30

**Authors:** Andrew T. McNutt, Fatimah Bisiriyu, Sophia Song, Ananya Vyas, Geoffrey R. Hutchison, David Ryan Koes

**Affiliations:** † Department of Computational and Systems Biology, University of Pittsburgh, Pittsburgh, Pennsylvania 15213, United States; ‡ The Neighborhood Academy, Pittsburgh, Pennsylvania 15206, United States; § Upper St. Clair High School, Pittsburgh, Pennsylvania 15241, United States; ∥ Taylor Allderdice High School, Pittsburgh, Pennsylvania 15217, United States; ⊥ Department of Chemistry, University of Pittsburgh, Pittsburgh, Pennsylvania 15213, United States; # Department of Chemical and Petroleum Engineering, University of Pittsburgh, Pittsburgh, Pennsylvania 15213, United States

## Abstract

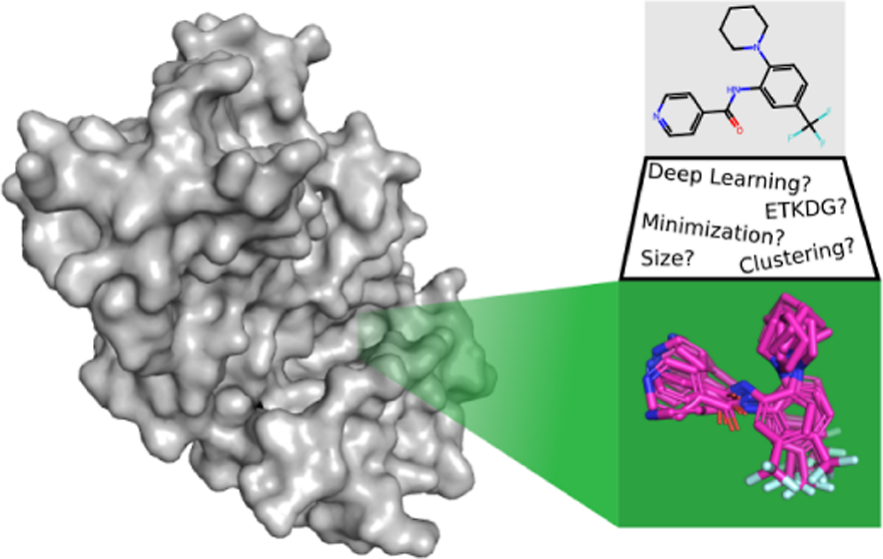

Conformer generation, the assignment of realistic 3D coordinates to a small molecule, is fundamental to structure-based drug design. Conformational ensembles are required for rigid-body matching algorithms, such as shape-based or pharmacophore approaches, and even methods that treat the ligand flexibly, such as docking, are dependent on the quality of the provided conformations due to not sampling all degrees of freedom (e.g., only sampling torsions). Here, we empirically elucidate some general principles about the size, diversity, and quality of the conformational ensembles needed to get the best performance in common structure-based drug discovery tasks. In many cases, our findings may parallel “common knowledge” well-known to practitioners of the field. Nonetheless, we feel that it is valuable to quantify these conformational effects while reproducing and expanding upon previous studies. Specifically, we investigate the performance of a state-of-the-art generative deep learning approach versus a more classical geometry-based approach, the effect of energy minimization as a postprocessing step, the effect of ensemble size (maximum number of conformers), and construction (filtering by root-mean-square deviation for diversity) and how these choices influence the ability to recapitulate bioactive conformations and perform pharmacophore screening and molecular docking.

## Introduction

Generating a three-dimensional conformation of a molecule from its topological representation (e.g., a SMILES string) is a fundamental first step of most structure-based approaches to drug discovery.^1,2^ Tasks such as 3D pharmacophore search,^3−5^ molecular docking,^6−9^ and (three-dimensional quantitative structure–activity relationship)^10^ all rely on the generation of a biochemically meaningful conformation. Traditionally, conformer generation algorithms have adopted either a systematic or a stochastic approach. Systematic approaches attempt to enumerate all reasonable values for rotatable bonds and thus often have difficulty scaling. Stochastic approaches use random sampling to make the search process more scalable. Distance geometry^11,12^ uses bond length, angle, and other, possibly knowledge-based,^13^ constraints to constrain the stochastic search space. More recently, machine learning has been used to either generate conformations directly^14−16^ or assist in the generation process otherwise (e.g., torsional sampling).^17−25^ Although there are multiple ways to evaluate the quality of a conformer generator,^1^ most relevant for structure-based drug discovery is the ability to produce a bioactive conformation. That is, a conformation close to the conformation found in a protein–ligand complex should be generated, even if it is not the lowest energy conformation. Both free^26^ and commercial^27^ conformer generators were evaluated for this task, and, provided that a sufficiently large ensemble is generated, most approaches succeed at identifying a low root-mean-square deviation (rmsd) (<2 Å) conformation. In particular, the open-source RDKit, which uses a stochastic distance geometry-based approach combined with experimental torsional-angle and ring geometry preferences (ETKDG),^13^ consistently performs as well as or better than other approaches such as Balloon, Confab, Frog2, Multiconf-DOCK, CREST, ConfGen, OMEGA, and MOE (see Friedrich et al.,^26^ Friedrich et al.,^27^ and Folmsbee et al.^28^), hence we limit our evaluation to RDKit as a representative of a conventional conformer generator. We note that the recently described Auto3D^29^ uses RDKit conformers as a starting point for optimizing with a modified version of the ANI-2x deep learning molecular potential,^30^ but this does not result in better performance than that of RDKit in the bioactive conformation identification task (see Figure S1).

The latest machine learning models have not been evaluated for their ability to generate bioactive conformations. Instead, they are mostly trained and evaluated on the GEOM^31^ data set, which contains 37 million conformers of more than 450,000 molecules with the goal of accurately representing, at the level of semiempirical density functional theory,^32^ the vacuum conformer–rotamer ensembles of these molecules using CREST.^33^ Deep generative models significantly outperform RDKit at this particular task, but an extended sampling and clustering approach using RDKit achieves a highly competitive performance.^34^ It is not clear that it is a fair comparison to compare methods that utilize different amounts of sampling,^35^ so here we evaluate RDKit and a deep generative model using identical sampling and ensemble formation criteria. As the direct molecular conformation generation (DMCG)^14^ was found to perform best at the task of reconstituting the ensembles of the GEOM-Drugs subset of GEOM, we evaluate it here at the task of bioactive conformation recovery. DMCG is an end-to-end generative model with a variational encoder/decoder architecture that learns all network parameters from the training data distribution, GEOM-Drugs.^31^ However, our main goal is not to extend previous evaluations^26,27,34^ but to explore the impact of various choices made in the conformer generation process, such as the size of the ensemble, the criteria for including conformers in the ensemble, and the use (or not) of energy minimization, on the ultimate end point of the common structure-based tasks of pharmacophore search and molecular docking.

## Methods

**Figure 1:**
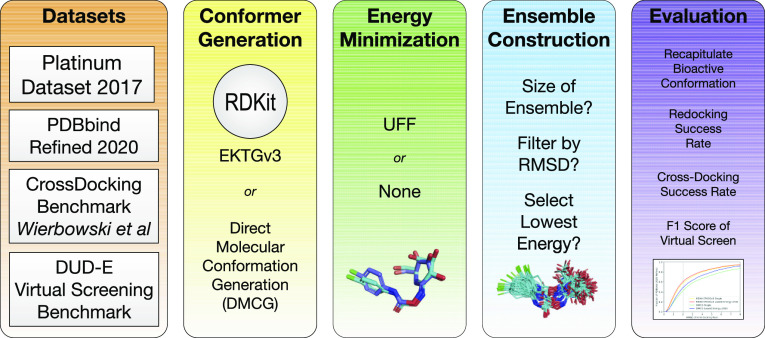
Overall workflow for conformer ensemble generation and evaluation in structure-based drug discovery tasks.

The overall workflow of our evaluation is shown in Figure 1. We evaluate a number of options for generating conformational ensembles from common data sets and evaluate them in two common structure-based tasks: pharmacophore search and molecular docking.

### Data Sets

**Figure 2:**
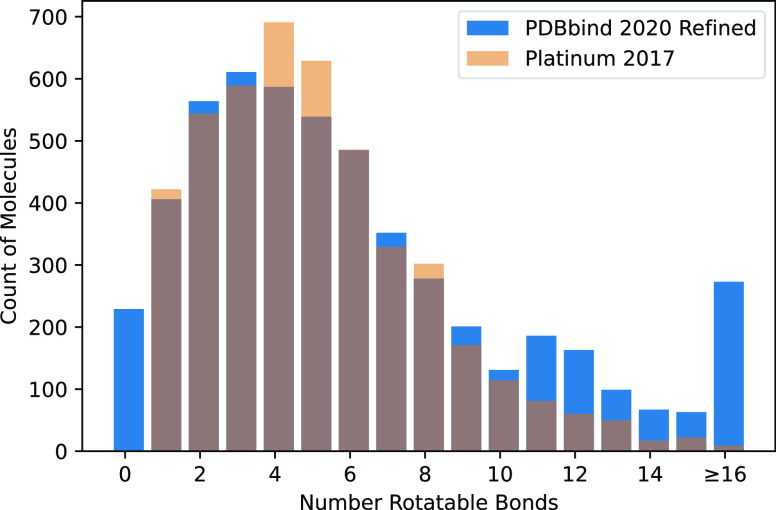
Histogram of rotatable bonds within our data sets.

In order to evaluate the ability of a conformer generator to produce a bioactive conformation, we use two data sets: Platinum 2017^27^ and the refined subset of PDBBind 2020.^36^ The PDBBind refined set curates high-quality protein–ligand structures from the Protein Data Bank with known binding affinities. Of the 5316 ligand structures in this set, 5313 could be processed by RDKit and are considered here (the remaining three all had a molecular weight of more than 900 Da). The Platinum data set was designed for conformer generation evaluations, and, in addition to considering the overall quality of a structure, it evaluates the quality of the fit of a ligand structure to the electron density map, ensuring that the included conformations are accurate. It also imposes more stringent filtering, such as not considering molecules with more than 16 rotatable bonds or fewer than one (Figure 2). While the Platinum data set provides a high-quality ground truth, the PDBbind data set contains more challenging (i.e., flexible) ligands.

For assessing the pharmacophore virtual screening performance, we use the database of useful decoys: enhanced (DUDE),^37^ which contains 102 protein targets, each with their own set of experimentally confirmed actives and (possibly putative) decoys. We note that while there are significant issues in using DUDE for training and evaluating machine learning approaches^38,39^ due to inherent biases in the data set construction that can result in misleading evaluations of generalization performance, the structure-based pharmacophore search we evaluate does not have these drawbacks as it is not fitting to the data. We note that the use of decoy molecules that are not experimentally validated in DUDE may result in false negatives, but this is not material for evaluating the trends in the virtual screening performance, which is our goal here. We also note that the actives in DUDE are experimentally validated against their target, which is essential when evaluating target-focused approaches and strongly preferable to using benchmarks, such as LIT-PCBA,^40^ where actives have uncertain mechanisms of action due to being identified in phenotypic screens.

For assessing molecular docking performance, we use the refined set of PDBBind 2020 for redocking evaluations and for cross-docking the data set of Wierbowski et al.^41^ Redocking evaluates docking a ligand to its cognate receptor while cross-docking docks a ligand to a similar, but not cognate, receptor. As the number of protein–ligand pairs in a cross-docking task grows combinatorially and we do not want to disproportionately weight targets with more structures, we randomly downsample the data set of Wierbowski et al’.^41^ to have at most 100 protein–ligand pairs for each of its 92 targets (63% of the targets require downsampling). Docking success is measured by calculating the rmsd between the top ranked docked pose and the crystal pose. In the case of cross-docking, the reference pose is determined by aligning the protein structure of the cognate receptor with the target receptor.

### Conformer Ensemble Generation

We generate RDKit conformers using the ETKDG version 3^12,13^ method of RDKit using default values and version 2022.03.1. This method combines distance geometry^11^ sampling with knowledge-based potentials to increase the efficiency of the algorithm without loss of accuracy (see Friedrich et al.^26^ and Figure S2). To generate DMCG^14^ conformers, we use the pretrained model Large_Drugs/checkpoint_94.pt with the recommended settings for drug-like molecules. We strip the input SMILES strings of stereochemistry information as this information is often missing in virtual screening data sets and we want to evaluate the ability of conformer generators to sample appropriate geometries (Figure S3 shows the relatively small contribution of stereochemistry in our evaluations). For both generators, we evaluate further refining generated conformations using the universal force field (UFF) molecular force field,^42^ as implemented by RDKit and default convergence criteria.

Consistent with previous evaluations,^26,27^ we generate ensembles with a maximum of 250 conformers. We consider different methods for subsetting the full ensemble, including unbiased sampling, energy ranking, and energy ranking with rmsd filtering.

### Pharmacophore Search

Pharmit^43^ is used to perform pharmacophore search on the DUDE benchmark. Search databases are built from the relevant conformational ensembles of the active and decoy compounds of each DUDE target. Due to the large number of conformers required, only RDKit conformer generation was evaluated for this task. The provided reference crystal structure is used to elucidate all possible interacting features (hydrogen bonds, hydrophobic interactions, charge interactions, and aromatic interactions) between the ligand and receptor. Interactions are identified using the built-in heuristics of Pharmit. From this set of interactions, all possible pharmacophores with at least three features are enumerated. As our goal is to evaluate the effect of different conformer ensembles on virtual screening and not elucidate the best single pharmacophore query, we screen all of the enumerated queries. We set a tolerance radius of 1.0 Å and no other constraints (e.g., direction) on each feature. Since Pharmit uses the sublinear time Pharmer^44^ algorithm, despite the combinatorial number of queries and many thousands of compounds, this can be done efficiently. We emphasize that this algorithm finds matches between the specified query and rigid conformers, and so, the quality of the ensemble is essential. As only matching, not ranking, is performed, classification metrics are the most appropriate choice to evaluate virtual screening performance in this context (i.e., without a ranking, it is not possible to calculate a meaningful AUC (area under the curve) of a receiver operating characteristic (ROC) or precision–recall curve). We use the F1 score, the harmonic mean of the precision and recall, and report the best F1 across all queries. Unlike an enrichment factor, the F1 score is a normalized quantity (ranges from 0 to 1) and so can be sensibly compared across different screens, and it encapsulates the goal of virtual screening to maximize the number of true positives (recall) while minimizing the number of false positives (higher precision).

### Molecular Docking

GNINA^7^ is used to perform molecular docking. GNINA is a fork of AutoDock Vina^45^ that uses a convolutional neural network protein–ligand scoring function^46^ to select and rank poses. Independent evaluations^47,48^ of GNINA have found it to have comparable performance to that of the commercial Glide software^49^ while outperforming other open-source docking programs such as smina.^50^ Poses are sampled using a Monte Carlo Metropolis algorithm that perturbs the rigid-body degrees of freedom (translation and rotation) and torsional degrees of freedom. The output docked poses therefore depend on the input conformation to determine the bond lengths and angles. However, internal torsions are completely randomized at the start of each Monte Carlo chain, so the result does not depend on the input torsions.

To assess the impact of the input conformation on docking results, we consider a single conformer and five conformer ensembles (larger ensembles were not considered due to the computational overhead of docking).

## Results

### Retrieval of Bioactive Conformers

**Figure 3:**
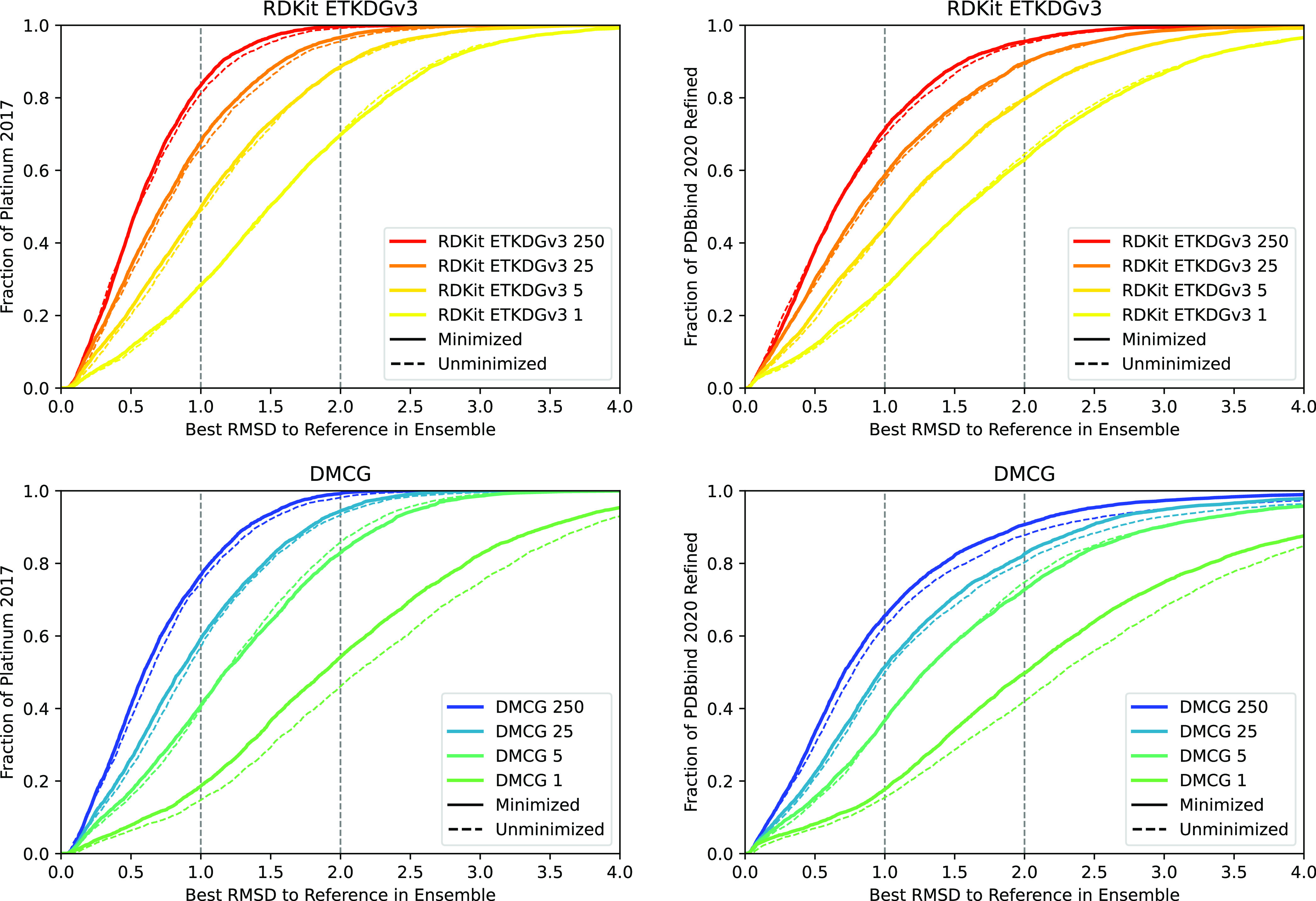
Fraction of the Platinum (left) and PDBbind2020 refined (right) data sets where a conformer within a specified threshold (*x*-axis) of the experimental structure is retrieved for both RDKit (top) and DMCG (bottom) for various-sized ensembles. Results for poses before (dashed line) and after (solid line) UFF minimization are shown.

**Figure 4:**
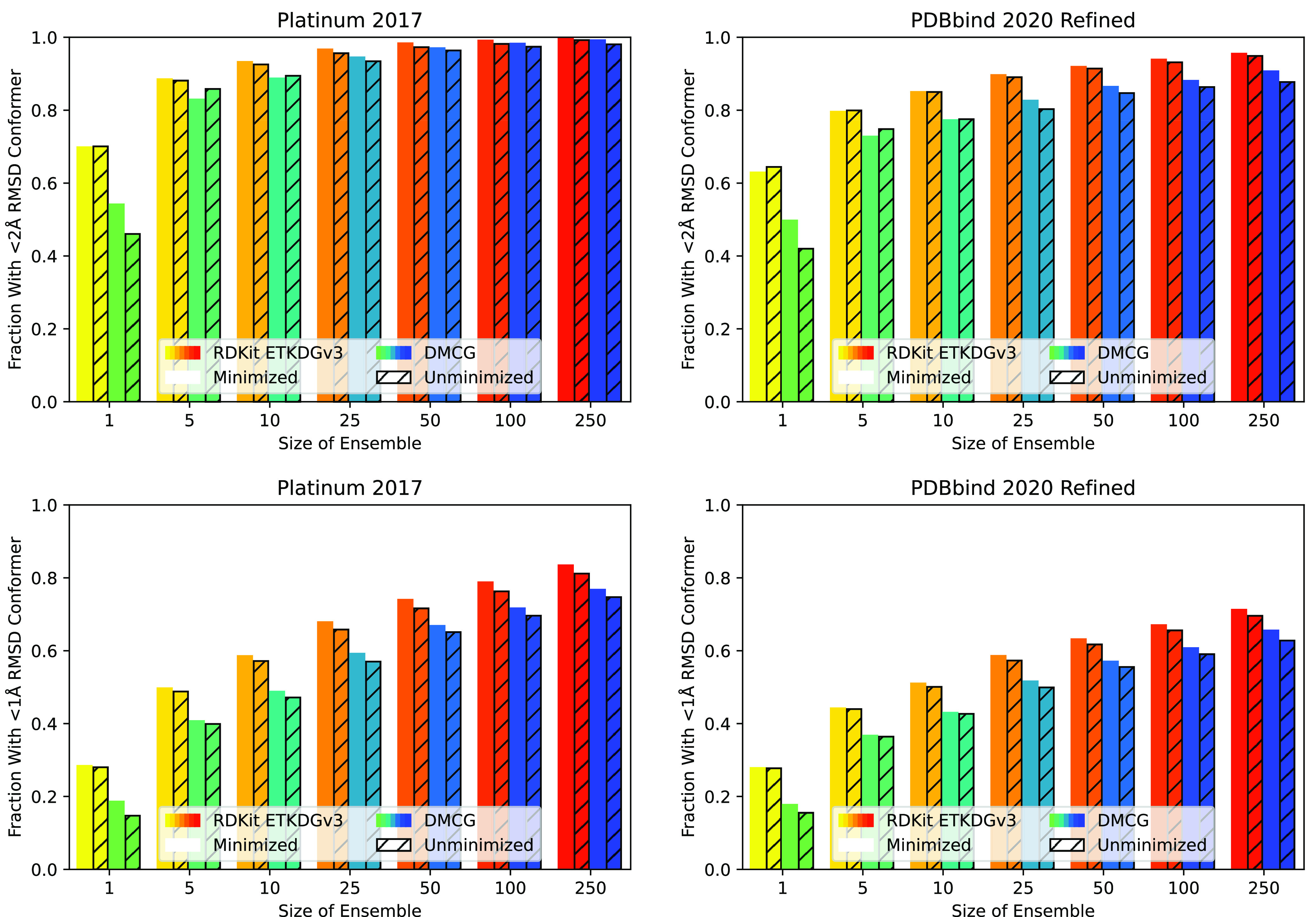
Fraction of the Platinum (left) and PDBbind2020 refined (right) data sets where a conformer within 2.0 Å (top) or 1.0 Å (bottom) rmsd of the experimental structure is retrieved for both RDKit and DMCG for various-sized ensembles. Results for poses before (hashed bars) and after (solid bars) UFF minimization are shown.

To compare the generated conformers to the experimental crystal structure, we use obrms from the Open Babel toolkit,^51^ which properly handles internal symmetries by reporting the lowest possible rmsd of any valid atom matching. In all cases, the minimized rmsd (-m option) is reported (i.e., the structures are optimally aligned before the rmsd). For a variety of ensemble sizes (number of samples of the specified method), we evaluate the fraction of the data set where a conformer exists within the ensemble for a specified rmsd threshold. That is, we consider the best possible rmsd across the ensemble. Results for a variable threshold are shown in Figure 3 with more ensemble sizes shown for two fixed thresholds, 1.0 and 2.0 Å in Figure 4. For reference, example structures at different rmsd values are shown in Figure S20. In general, we find that RDKit consistently matches or outperforms DMCG at conformer retrieval at every rmsd threshold (a more direct visual comparison is found in Figure S4). This advantage is greater on the PDBbind data set as RDKit better handles larger, more flexible ligands (Figure S5). The larger, more flexible ligands in the PDBbind data set result in consistently lower retrieval rates for both methods, but the trends between the two data sets are consistent. Energy minimization has a small, not always beneficial, effect that is more pronounced and generally beneficial for DMCG. Unless otherwise specified, we limit ourselves to evaluating minimized conformers for the remainder of our analysis.

**Figure 5:**
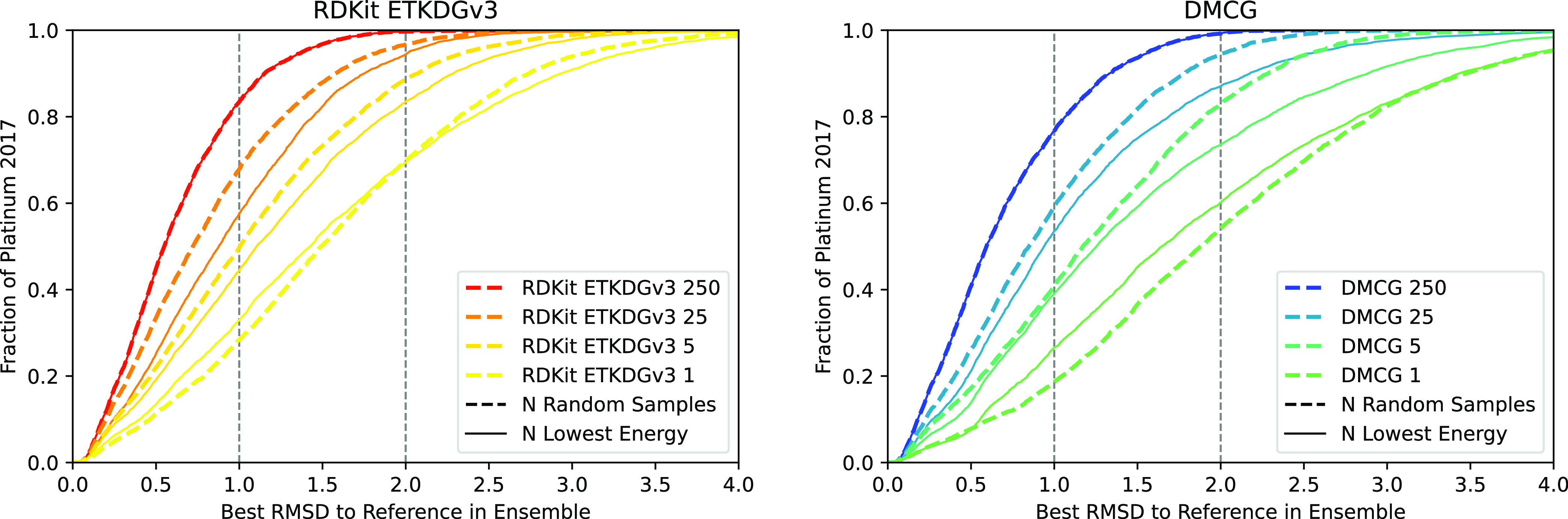
Comparison of RDKit ETKDGv3 (left) and DMCG (right) on the Platinum 2017 data set when evaluated on different ensemble sizes where the ensemble is constructed by selecting the *N* lowest-energy-minimized conformers are selected from an ensemble of 250. Results for the PDBbind refined data set are shown in Figure S6 and exhibit a similar trend.

**Figure 6:**
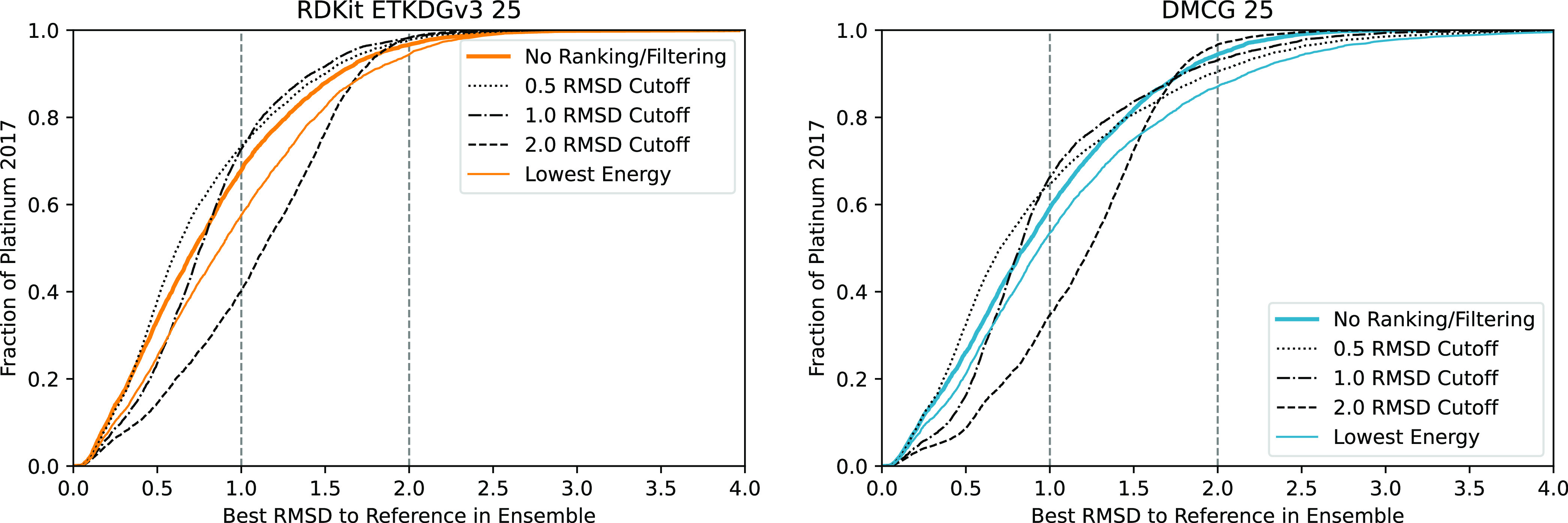
Evaluation of different methods of constructing an ensemble of 25 conformers selected from a 250 conformer ensemble generated using RDKit ETKGDv3 (left) or DMCG (right) on the Platinum 2017 data set. PDBbind refined results are shown in Figure S8.

Generating a larger ensemble will monotonically increase the likelihood of retrieving a bioactive conformation, but for efficiency reasons, it is desirable to generate smaller ensembles. As shown in Figures 5 and S6, selecting the lowest energy poses from a larger ensemble does not improve the retrieval of bioactive conformations with the exception of reducing down to a single conformer. This is due to the lack of geometric diversity of subsets chosen using only energy as a criterion, as well as the energy evaluation reflecting an isolated ligand, neglecting nonbonded dispersion interactions with the surrounding protein. In Figures 6 and S7–S9, we show the effect of imposing an rmsd cutoff when selecting conformers. In this case, the conformers of the full 250 conformer ensemble are sorted by increasing energy, and we greedily add conformers to the selected subset only if their rmsd to every already selected conformer is greater than an rmsd threshold. This approach results in an improved retrieval rate relative to unbiased sampling or lowest energy selection when the rmsd threshold used to select conformers is similar to the rmsd cutoff used to classify a conformer as matching the experimental structure. For example, selecting 25 RDKit conformers for Platinum 2017 using an rmsd of 1.0 results in ensembles that contain a conformer within 1.0 rmsd of the true conformer 72.7% of the time, compared to 67.8% when unbiased sampling is used and 57.6% when the 25 lowest-energy conformations are selected. However, if the rmsd criteria for determining what qualifies as a matching conformation deviate significantly from the rmsd threshold used to select the subset (either lower or higher), unbiased sampling can outperform the filtered subsets. Finally, we note that in our analysis, the bioactive conformation depends on the structure of a receptor, which is hidden information from the conformer generator, but we observe similar trends in retrieval rates when we evaluate using a curated subset^28^ of the Crystallography Open Database^52^ (see Figure S10), which contains a broader array of single-molecule experimental structures.

### Conformer Ensemble Effect on Pharmacophore Search

Although retrieval of bioactive conformations within a conformational ensemble is clearly desirable, it is not clear whether such an analysis is sufficient to determine the best approach for constructing conformational ensembles for structure-based tasks. To more directly address this question, we consider rigid pharmacophore matching against differently sized conformer ensembles. We consider generating conformers with RDKit and then unbiased sampling from energy minimized poses, as well as sorting by energy and then filtering by a specified rmsd threshold as this approach was found to be most effective at retrieving bioactive conformations. This threshold specifies the minimum distance between two conformers in the ensemble.

**Figure 7:**
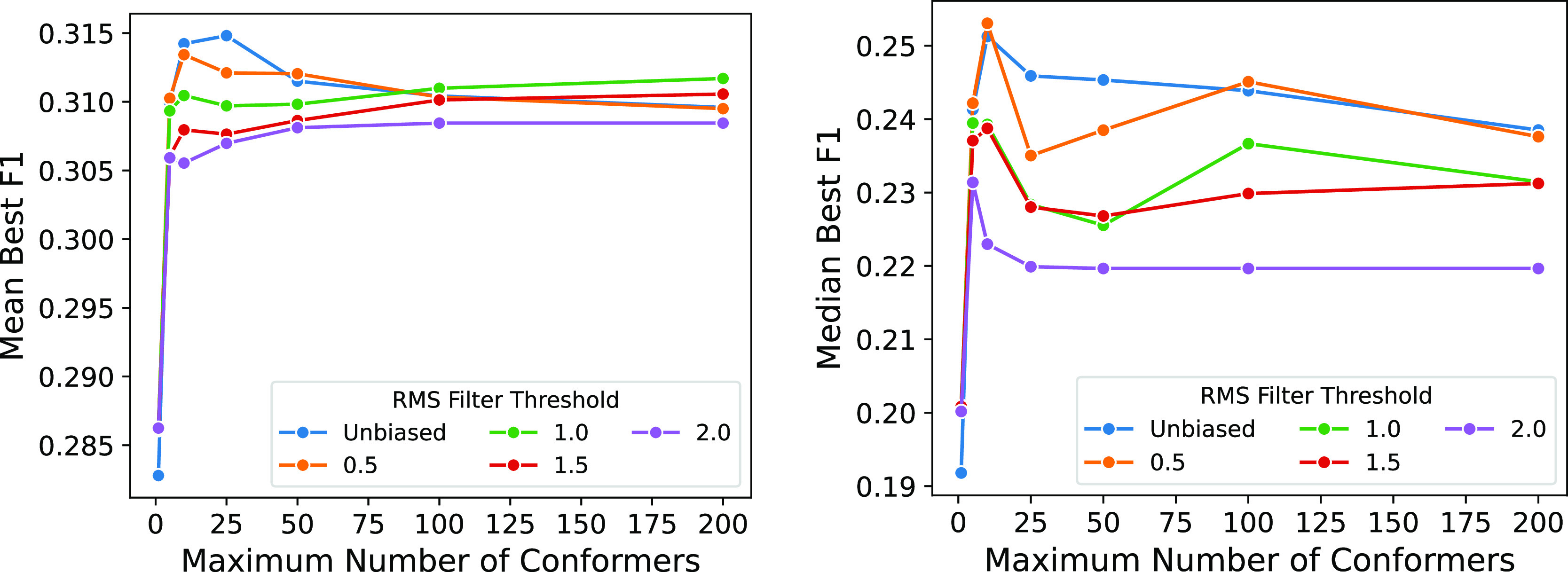
Average and median best F1 score achieved from pharmacophore search across the 102 DUDE targets as the maximum number of conformers allowed in the library is varied.

The overall average effect on F1 score as the rmsd threshold and maximum number of conformers in a generated ensemble are varied is shown in Figure 7. For smaller ensembles (e.g., <10 conformers), using lower energy poses filtered by rmsd provides the best average performance. For moderately sized ensembles, a larger rms threshold reduces performance. Larger rms thresholds result in significantly smaller ensembles (e.g., filtering at a 2 Å threshold results in a reduction from 250 conformers to an average of only 6 conformers per molecule, see Figure S11), hence larger amounts of filtering result in reduced performance and are relatively insensitive to increasing the maximum allowed number of conformers. While increasing the number of conformers can only increase the recall of known actives, it can also reduce the precision (i.e., increase the number of false positives due to more inactive compounds matching the pharmacophore). This leads to a reduction in average performance as the maximum size of the ensemble is increased with minimal filtering.

**Figure 8:**
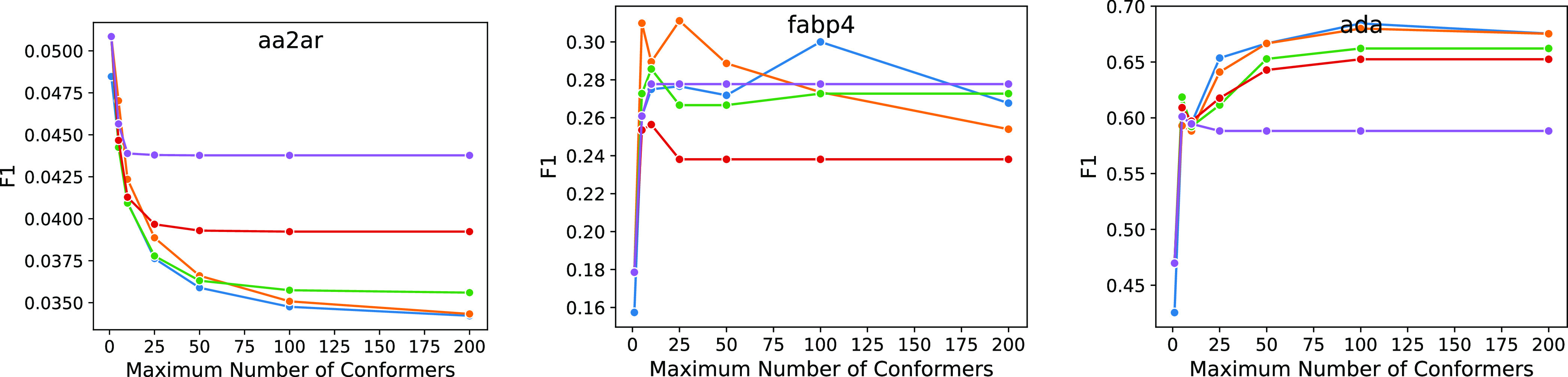
Best F1 score achieved from the pharmacophore as the maximum number of conformers allowed in the library is varied for three distinctly different targets. Note that the *y*-axis scales differ to better illustrate the trends. Individual F1 plots are shown for all targets in Figures S12 and S13.

**Figure 9:**
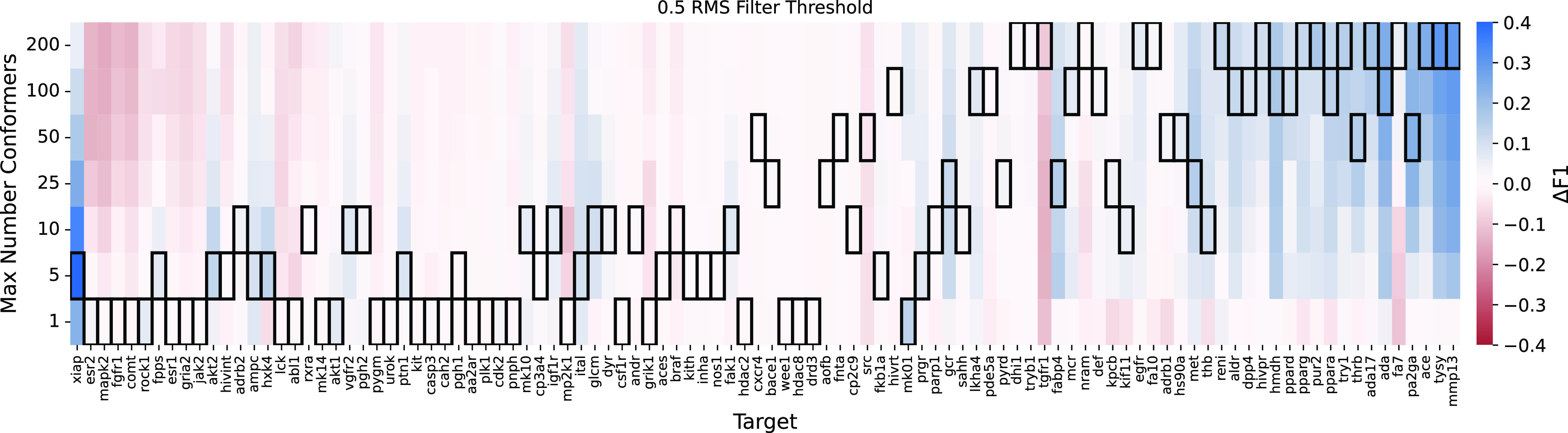
For each DUDE target, the difference in the best achieved F1 score relative to the best F1 achieved by unbiased sampling of a single conformation is shown for different numbers of maximum allowed conformations. Conformers are selected by sorting by energy and then filtered by an rms threshold of 0.5 Å. Targets are sorted by the slope of the best fit line through the conformer/F1 data. Box outlines highlight the choice of maximum number of conformers that provides the highest F1 score.

The average effect size shown in Figure 7 is small; however, as shown in Figures 8 and 9, the average trends hide a wide array of responses to changes in conformational ensemble size, and the effect of ensemble size can be significant and varied. For the 0.5 Å rms filtered set, there are 29 of the 102 targets where the best F1 score is achieved using a single conformer compared to 16 where the best F1 score is achieved using an ensemble of 200 conformers. For the majority of targets (68), the best F1 score requires 25 or fewer conformers, and for cases where more conformers are preferred, the improvement over smaller ensembles is often minimal.

**Figure 10:**
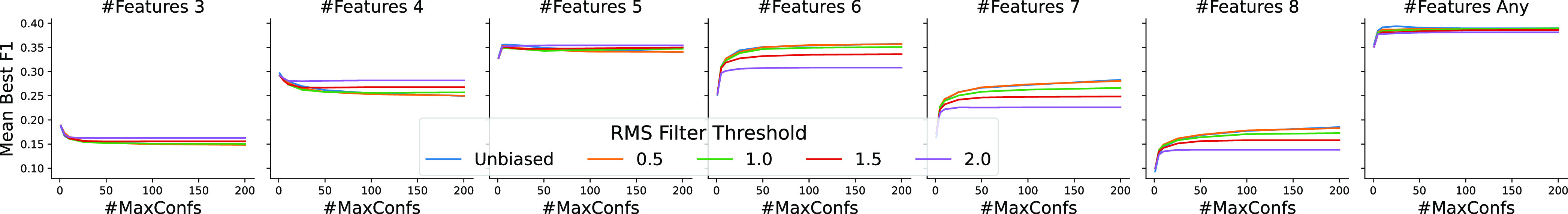
Best F1 score achieved from pharmacophores with a specified number of features for different choices of conformer ensembles. Only a subset of 63 targets is evaluated as the remaining targets have fewer than 8 interaction features to select from. For reference, the mean F1 when the number of features is not fixed (“Any”) is shown.

For the previous analysis, we consider only the best performing (by F1 score) pharmacophore query, essentially assuming the pharmacophore query was designed by an omniscient oracle, in order to separate the issue of pharmacophore elucidation from the effect of the choice of conformer ensemble. However, it is instructive to consider the change in trends if the oracle is restricted to pharmacophore queries with a fixed number of features, as shown in Figure 10. As the number of features is increased, the specificity of the query increases, and the number of matches decreases. For low-specificity queries, small ensembles maximize the F1 as they counterbalance the lack of specificity, while high-specificity queries benefit from large ensembles. In order to achieve the best F1 performance, a balance of both query specificity and ensemble size is needed.

### Conformer Ensemble Effect on Molecular Docking

While screening approaches that use rigid conformer ensembles are used for some structure-based screens, many approaches, such as molecular docking, treat input molecules as partially flexible, and the rotatable bonds are explicitly optimized. While it might seem that conformational ensembles are unnecessary for these approaches, exploring the nontorsional degrees of freedom may still have some value. For example, the popular Glide docking program explicitly considers nontorsional degrees of freedom by sampling alternative ring conformations and nitrogen inversions.^49^

**Figure 11:**
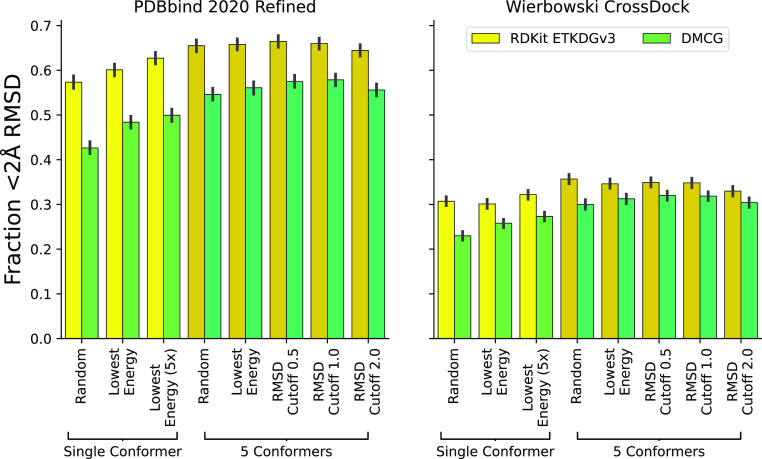
Effect of using different input conformer ensembles on docking performance as measured by the fraction of systems where a low (<2 Å) rmsd pose is identified as the top ranked docked pose. Error bars indicate the 95% confidence interval determined from 1000 bootstraps. Figures S16–S19 show similar trends for different choices of the rmsd cutoff.

We explore the impact of providing different conformer ensembles as input on docking performance in Figure 11. RDKit consistently outperforms DMCG at recapitulating low (<2 Å) rmsd poses. We highlight performance at a 2 Å cutoff due to its frequent use in docking evaluations.^53,54^ Similar trends are observed for different choices of cutoffs (Figures S16–S19). Using the lowest-energy sampled conformation (from an ensemble of 250 conformers) performs better than a randomly sampled conformation for both methods. DMCG in particular benefits from using an energy-minimized conformation (Figure S16) as energy minimization fixes nonstandard geometries. Interestingly, using an ensemble of five conformations outperforms a single conformation, even when the amount of Monte Carlo sampling during docking increased 5× to match the additional sampling performed using the ensemble input. This difference is statistically significant (*p*-value <0.001), although there is not always statistically significant difference between a randomly selected ensemble and seemingly more principled methods (with the exception of imposing a 2.0 Å rmsd cutoff, which can reduce the ensemble size to less than five in some cases). These results point to the need to go beyond sampling only the torsional space when docking, but also indicate that docking performance can be improved simply by providing conformational ensembles to dock; it may not be necessary to change the internal docking sampling algorithm.

## Discussion

We evaluated two conformer generators, the popular RDKit ETKDG method and the current state-of-the-art deep generative DMCG method, with a focus on exploring the effect of different choices in constructing conformer ensembles for structure-based drug discovery tasks. From the exercise, we draw several conclusions.

### Conventional Methods Remain Preferable to Deep Generative Models for Practical Applications

We find that the classical RDKit method is generally superior to DMCG at recovering bioactive conformations (Figures 3–5) and providing conformations suitable for docking (Figure 11). Given the current rate of progress, it is likely that newer deep generative methods will be able to outperform RDKit, although this may require adapting the metrics for which these methods are trained for. We speculate that the performance gap between these two methods is due to DMCG being trained to maximize coverage of the GEOM-QM9 and GEOM-Drugs sets. That is, it prefers to sample uniformly from the space of reasonable conformers, while RDKit may sample from something closer to a Boltzmann distribution. This speculation is supported by reports that resampling RDKit ensembles using clustering can substantially improve its coverage metric on GEOM-QM9 and GEOM-Drugs.^34^

### Energy Minimization Is a Valuable Postprocessing Step

Energy minimizing-generated conformers generally improve their ability to recapitulate bioactive conformers (Figures 3 and 4), and selecting the lowest-energy conformer generally performs better than a random conformer (Figures 5, S6, 7, and 11). Energy minimization is particularly important for improving the quality of DMCG-generated conformers, while the built-in geometric and knowledge-based constraints of RDKit’s ETKDG v3 algorithm need less refinement.

### Selecting Only the Lowest-Energy Conformers Is Not Sufficient to Achieve the Best Retrieval of Bioactive Conformations

While energy minimization improves poor-quality geometries, selecting the lowest-energy poses from a larger ensemble does not improve the retrieval of bioactive conformations when selecting more than a single conformer (Figure 5). The structure of a receptor and the nonbonded interactions with the ligand or solvent are hidden information from the conformer generation, and thus, the energy from an isolated molecule calculation, whether with UFF or a dispersion-corrected semiempirical method such as CREST-GFN2, is less useful than geometric diversity via rmsd clustering. Similarly, machine learning methods such as DMCG, which train on CREST-generated ensembles may reflect a bias toward low-energy and not bioactive conformations.^28^

### Larger Ensembles Are Not Always Better

Larger ensembles will always have higher likelihood at sampling a bioactive conformation, but for reasonable rmsd thresholds, the point of diminishing returns is achieved relatively quickly. It is not necessary to generate many hundreds of conformations to achieve nearly perfect recall within the 2 Å rmsd (Figures 5 and S6). Furthermore, in screening tasks, generating larger ensembles can decrease performance (Figures 7–9) due to increasing the number of false positives. Although the best observed ensemble size for maximal pharmacophore screening performance varied dramatically (from one to the maximum of 200, Figure 9), the diminishing returns in screening performance and increased computational complexity incurred by increasing the ensemble size suggest a reduced conformational ensemble of less than 25 conformers, which is likely sufficient for most structure-based screening tasks that rely on conformational sampling.^a^ We emphasize that this recommendation is not primarily motivated by the reduced computational demands of generating and screening more conformers but by the potential decrease in accuracy that arises when generating more conformers, which increases the false positive rate faster than the true positive rate.

### Filtering for Structural Diversity Can Enhance the Performance of a Given Ensemble Size

Unsurprisingly, when constructing a smaller ensemble from a larger ensemble, there is a benefit to increasing diversity by filtering conformations by their respective rmsds. When the goal is to recapitulate a bioactive conformer, the optimal choice of filtering threshold is strongly related to what rmsd value is used to determine a sufficiently close match (Figure 6). When a pharmacophore search is performed, more stringent thresholds are required and are especially important when smaller ensembles are used (Figure 7).

### Conformer Ensembles Are Useful Even for Tasks That Sample Torsional Degrees of Freedom

Finally, we note that conformational sampling is often focused on the sampling of torsions (indeed, some methods only sample torsions^22−25^). However, the nontorsional degrees of freedom also matter and can materially affect docking performance, as illustrated in Figure 11, which shows that providing an ensemble conformer with different nontorsional parameters improves docking performance over that with providing a single conformer.

## Supporting Information Available

Comparison of ETKDGv3 in RDKit to Auto3D on the PDBbind2020 refined dataset, comparison of regular distance geometry to ETKDGv3 in RDKit on the Platinum and PDBbind2020 refined datasets, evaluation of the effect of including stereochemistry in the provided SMILES string, evaluation of RDKit ETKDGv3 and DMCG on the Platinum and PDB bind2020 refined datasets, comparison of RDKit ETKDGv3 and DMCG on the Platinum and PDB bind2020 refined datasets as a function of the number of rotatable bonds, comparison of RDKit ETKDGv3 and DMCG on the PDBbind2020 refined dataset when the N lowest-energy minimized conformers are selected, evaluation of different methods of selecting a subset of 5 conformers from a 250 conformer ensemble, evaluation of retrieval of conformations from the Crystallography Open Database, distribution of the average number of conformers per a molecule for each DUDE target, relationship between best F1 score achieved through pharmacophore search and the maximum number of conformers allowed, difference in best achieved F1 score relative to the best F1 achieved by unbiased sampling of a single conformation, number of targets with best F1 score for a given choice of maximum conformers and filtering threshold, effect of using an energy-minimized pose from RDKit or DMCG as input for docking, effect of using the lowest-energy pose from RDKit or DMCG versus a randomly selected pose as input for docking, effect of different criteria for constructing an ensemble of five low-energy conformers, effect of using different input conformer ensembles on docking performance as measured by the fraction of systems, and examples of aligned conformations spanning different RMSDs for a selection of molecules with different numbers of rotatable bonds (PDF)

## Notes

Instructions and scripts for reproducing all the described analyses can be found at https://github.com/dkoes/conformer_analysis under an open-source Apache license.

## Footnotes

a ChatGPT 4.0, when asked the right number of conformers for such tasks, suggests 100–500 conformers per a molecule, suggesting that the data presented here runs counter to prevailing sentiment.
