# Supplementary material for: Conformer Generation for Structure-Based Drug Design: How Many and How Good?
Source: J Chem Inf Model. 2023 Oct 30;63(21):6598–607. doi: 10.1021/acs.jcim.3c01245 (PMC10647020; doi:10.1021/acs.jcim.3c01245)
Supplement: Supplementary file 1 — ci3c01245_si_001.pdf [file ci3c01245_si_001.pdf]

Supporting Information: Conformer Generation for Structure Based Drug Design: How Many and How Good?

Andrew T. McNutt,[†] Fatimah Bisiriyu,[‡] Sophia Song,[¶] Ananya Vyas,[§] Geoffrey R. Hutchison,[*,‖,⊥] and David Ryan Koes[*,#]

†*Department of Computational and Systems Biology, University of Pittsburgh, Pittsburgh, PA*

‡*The Neighborhood Academy, Pittsburgh, PA, 15206*

¶*Upper St. Clair High School, Pittsburgh, PA, 15241*

§*Taylor Allderdice High School, Pittsburgh, PA, 15217*

‖*Department of Chemistry, University of Pittsburgh, Pittsburgh PA, 15213*

⊥*Department of Chemical and Petroleum Engineering, University of Pittsburgh, Pittsburgh, PA, 15213*

#*Department of Computational and Systems Biology, University of Pittsburgh, Pittsburgh, PA, 15213*

E-mail: geoffh@pitt.edu; dkoes@pitt.edu

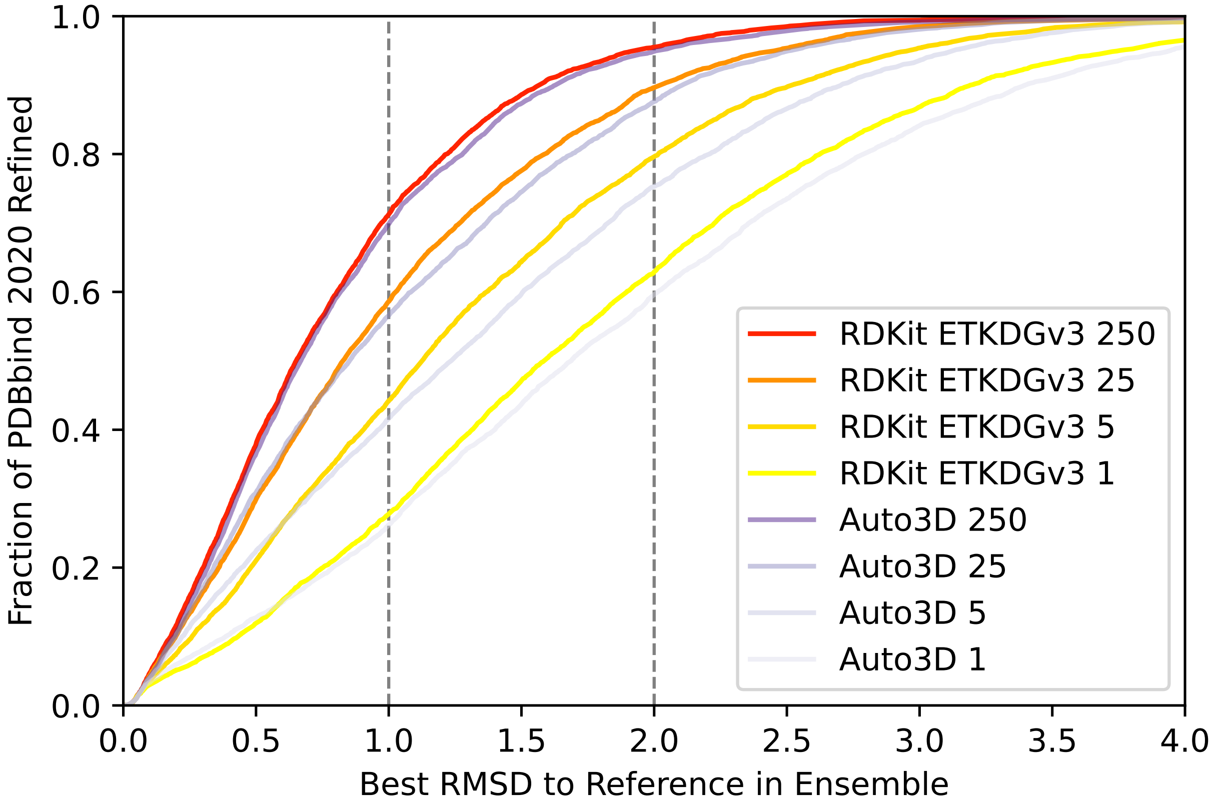

Figure S1: Comparison of ETKDGv3 in RDKit to Auto3D on the PDBbind2020 Refined dataset. Auto3D was run using the commandline `auto3D.py refined.smi --k 250 --max_confs 250 --threshold 0 --window 1000000` which disables Auto3D's built-in diversity clustering (RMSD threshold of zero) and energy filtering to make it comparable to the RDKit workflow. Auto3D failed to generate a conformer for about 3% of the PDB refined ligands; these ligands were also omitted for RDKit for this analysis.

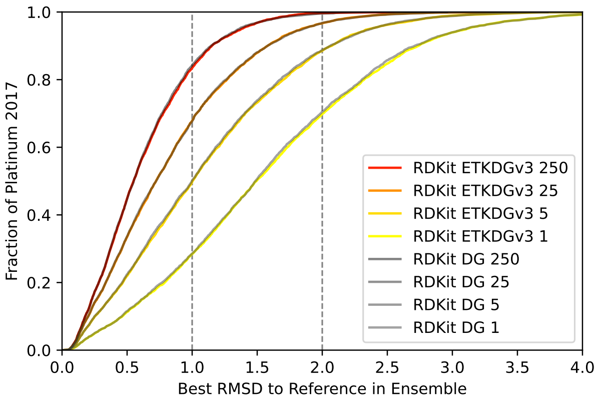

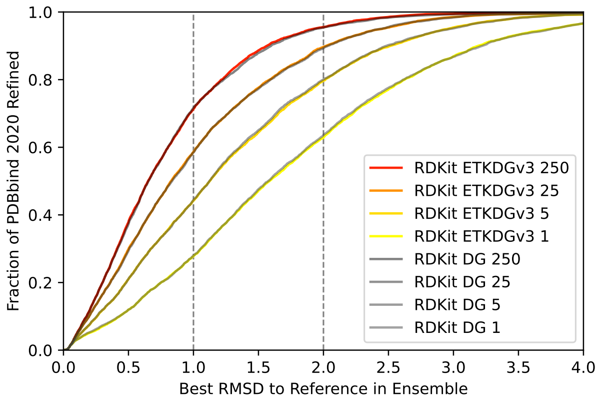

Figure S2: Comparison of regular distance geometry (DG) to ETKDGv3 in RDKit on the Platinum (left) and PDBbind2020 Refined (right) datasets. They exhibit nearly identical performance.

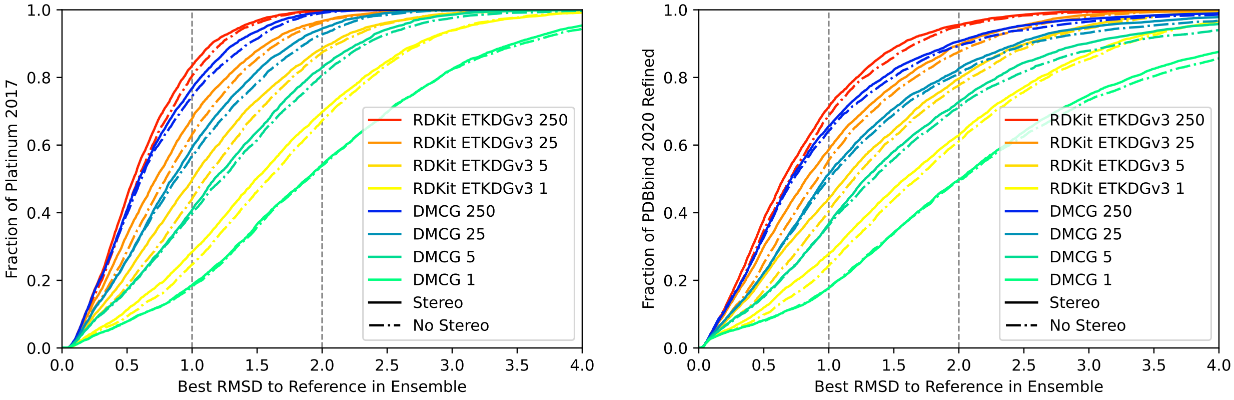

Figure S3: Evaluation of the effect of including stereochemistry in the provided SMILES string on the Platinum (left) and PDBbind2020 Refined (right) data sets for both RDKit and DMCG. Including this information results in a slight improvement, but not as much as using a better method or sampling more.

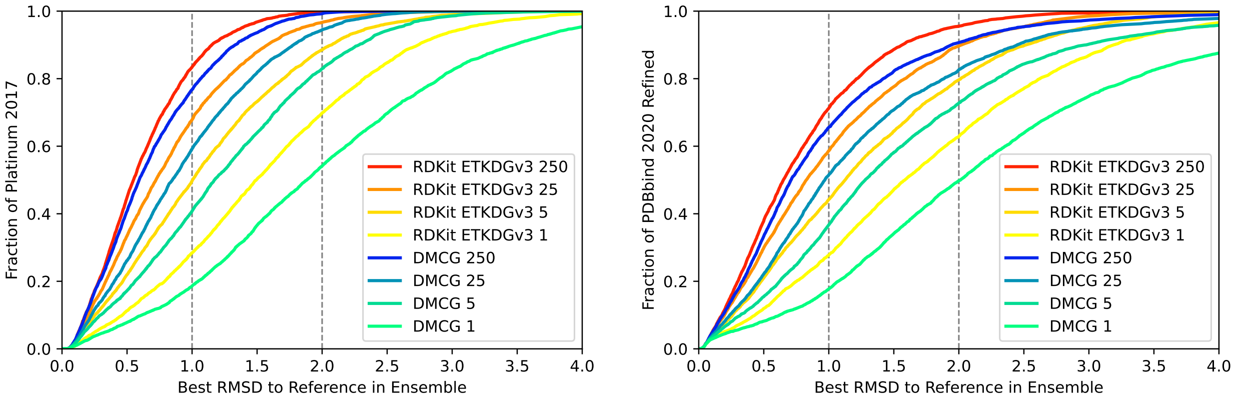

Figure S4: Evaluation of RDKit ETKDGv3 and DMCG on the Platinum (left) and PDB-bind2020 Refined (right) datasets. RDKit consistently outperforms DMCG.

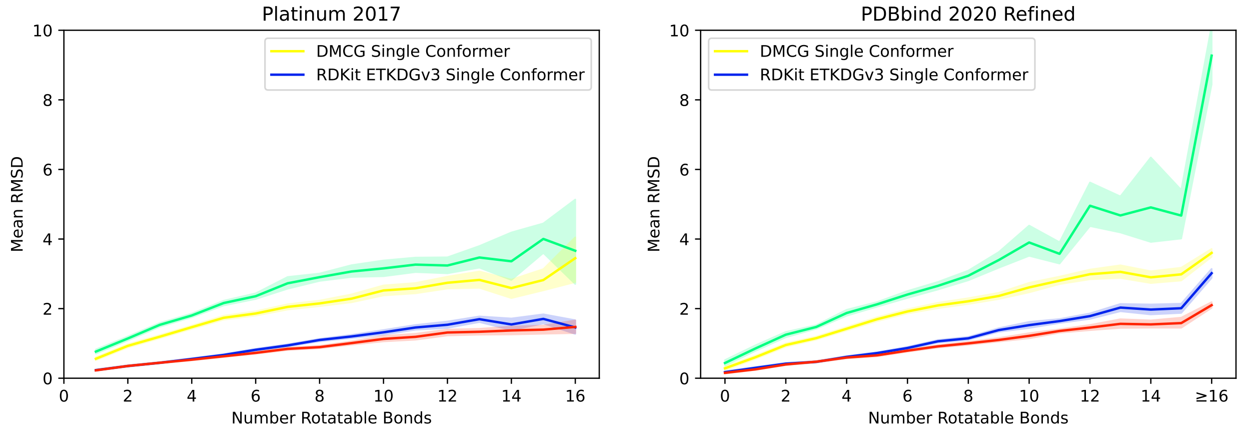

Figure S5: Comparison of RDKit ETKDGv3 and DMCG on the Platinum (left) and PDB-bind2020 Refined (right) datasets as a function of the number of rotatable bonds. RDKit consistently outperforms DMCG and its advantage increases with the flexibility of the target molecule.

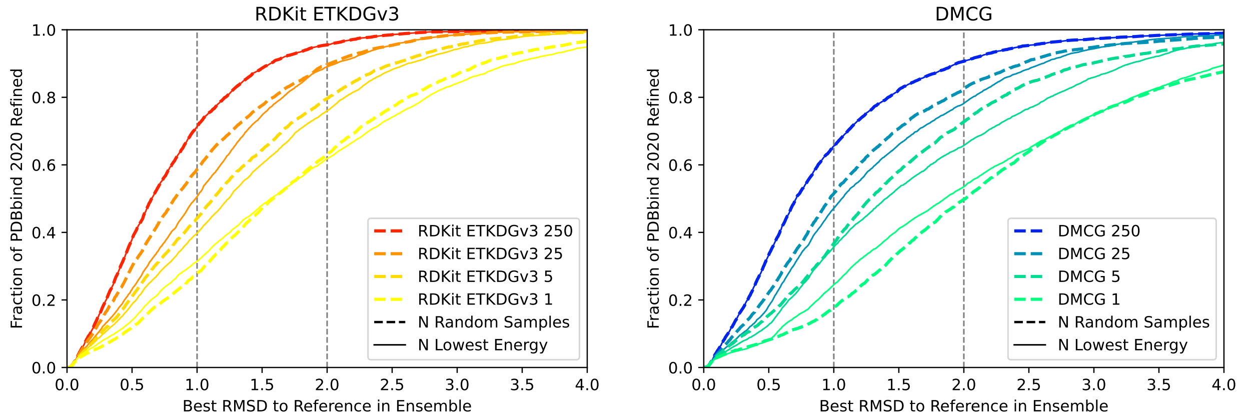

Figure S6: Comparison of RDKit ETKDGv3 (left) and DMCG (right) on the PDBbind2020 Refined dataset when the N lowest energy minimized conformers are selected from an ensemble of 250. With the exception of the single conformer (N=1) ensemble, selecting the lowest energy conformations is detrimental to recovering bioactive conformations.

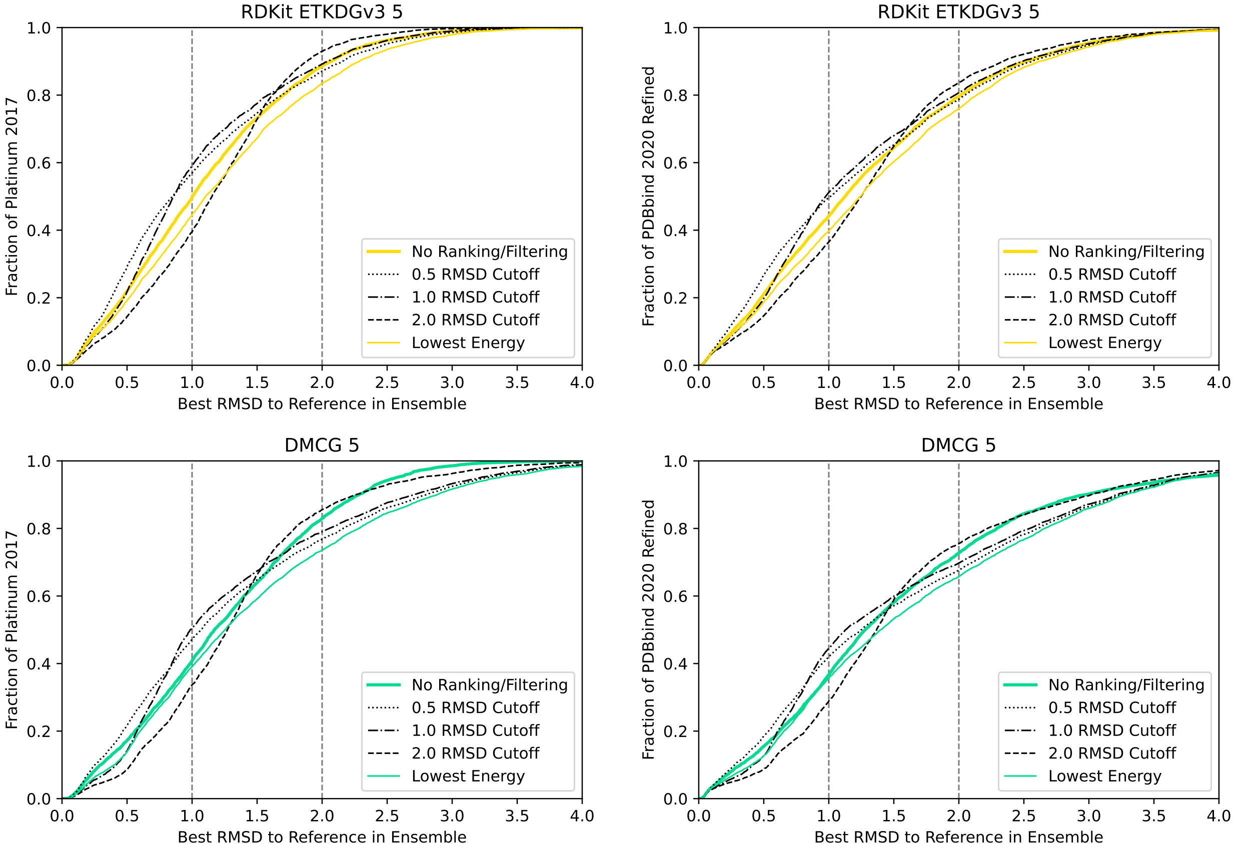

Figure S7: Evaluation of different methods of selecting a subset of 5 conformers from a 250 conformer ensemble generated using RDKit ETKGDv3 (left) or DMCG (right) on the Platinum 2017 (top) and PDBbind Refined (bottom) datasets. Imposing an RMSD cutoff is achieves the best performance when the RMSD to the experimental structures is considered a match at the same value as the cutoff.

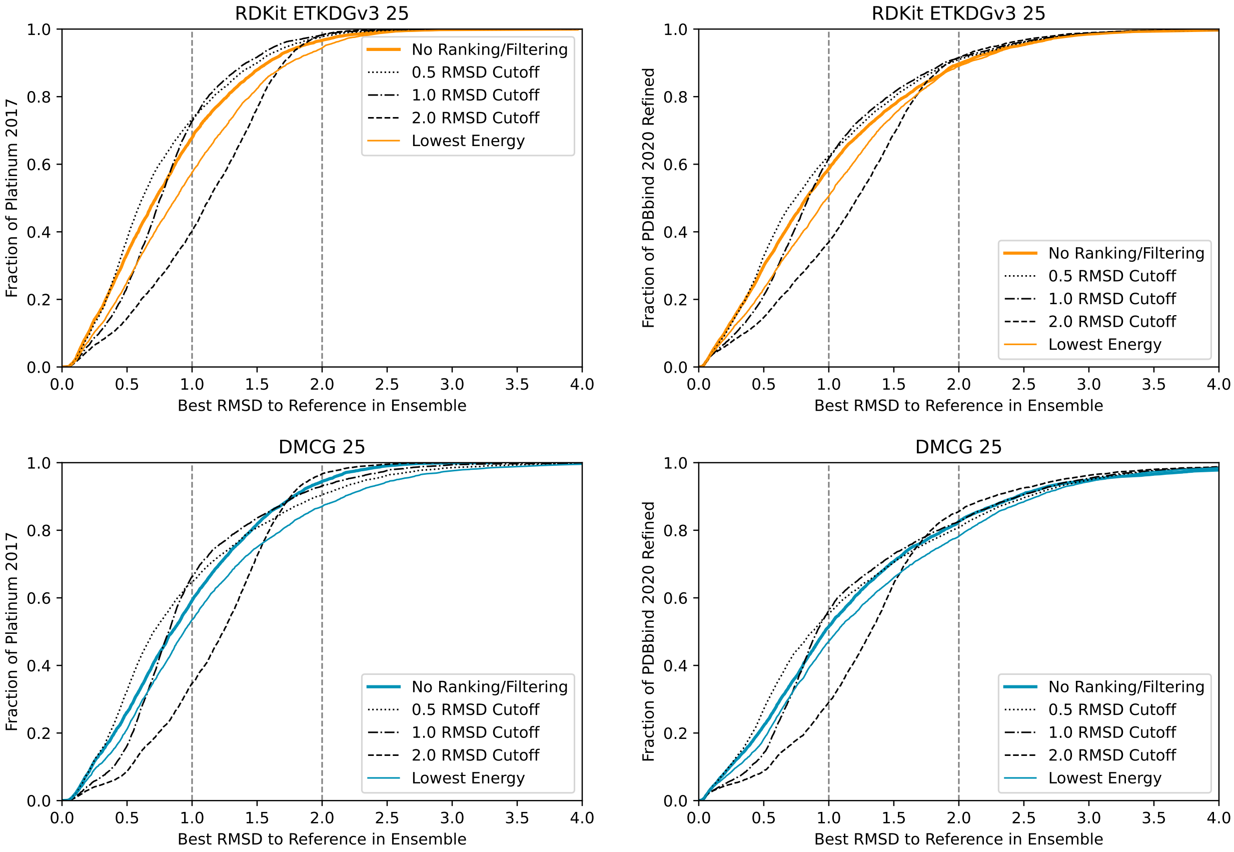

Figure S8: Evaluation of different methods of selecting a subset of 25 conformers from a 250 conformer ensemble generated using RDKit ETKGDv3 (left) or DMCG (right) on the Platinum 2017 (top) and PDBbind Refined (bottom) datasets. Imposing an RMSD cutoff is achieves the best performance when the RMSD to the experimental structures is considered a match at the same value as the cutoff.

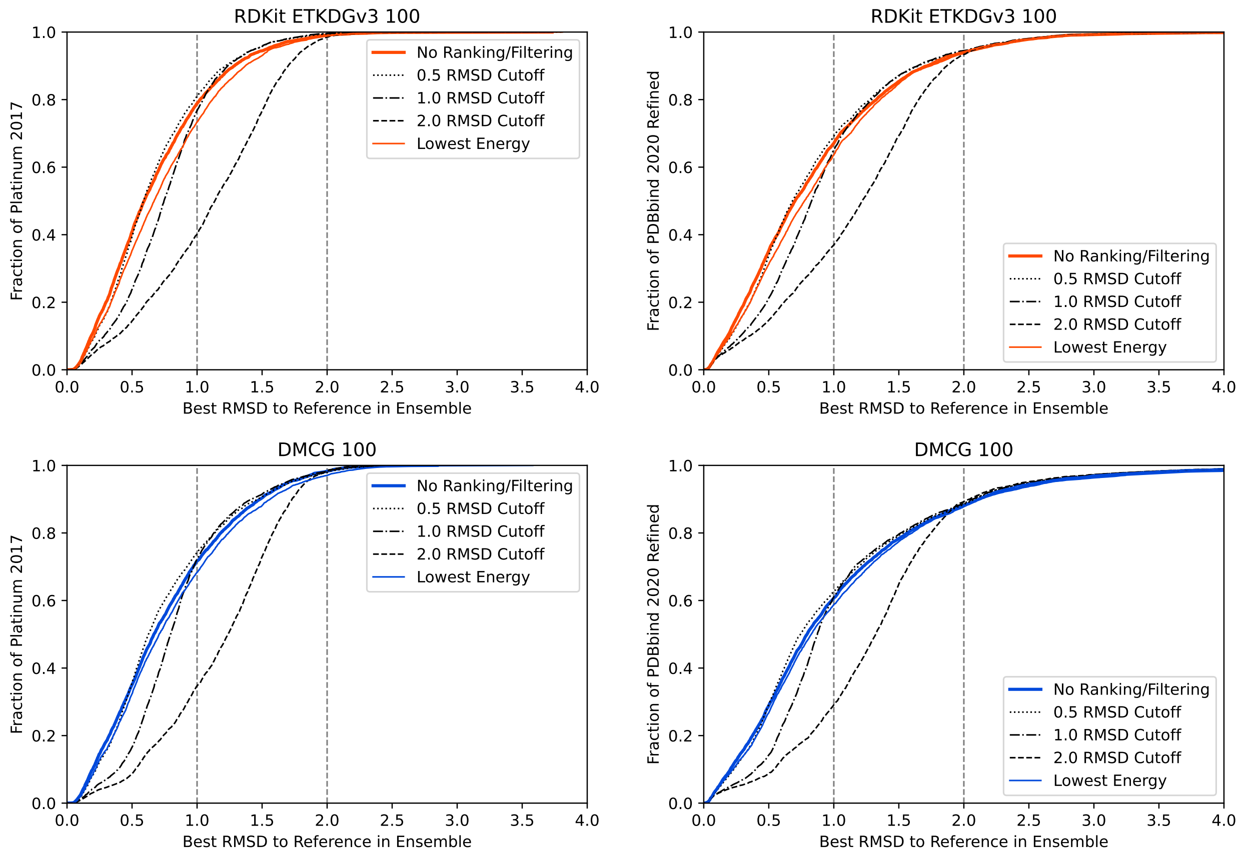

Figure S9: Evaluation of different methods of selecting a subset of 100 conformers from a 250 conformer ensemble generated using RDKit ETKGDv3 (left) or DMCG (right) on the Platinum 2017 (top) and PDBbind Refined (bottom) datasets. Imposing an RMSD cutoff is achieves the best performance when the RMSD to the experimental structures is considered a match at the same value as the cutoff.

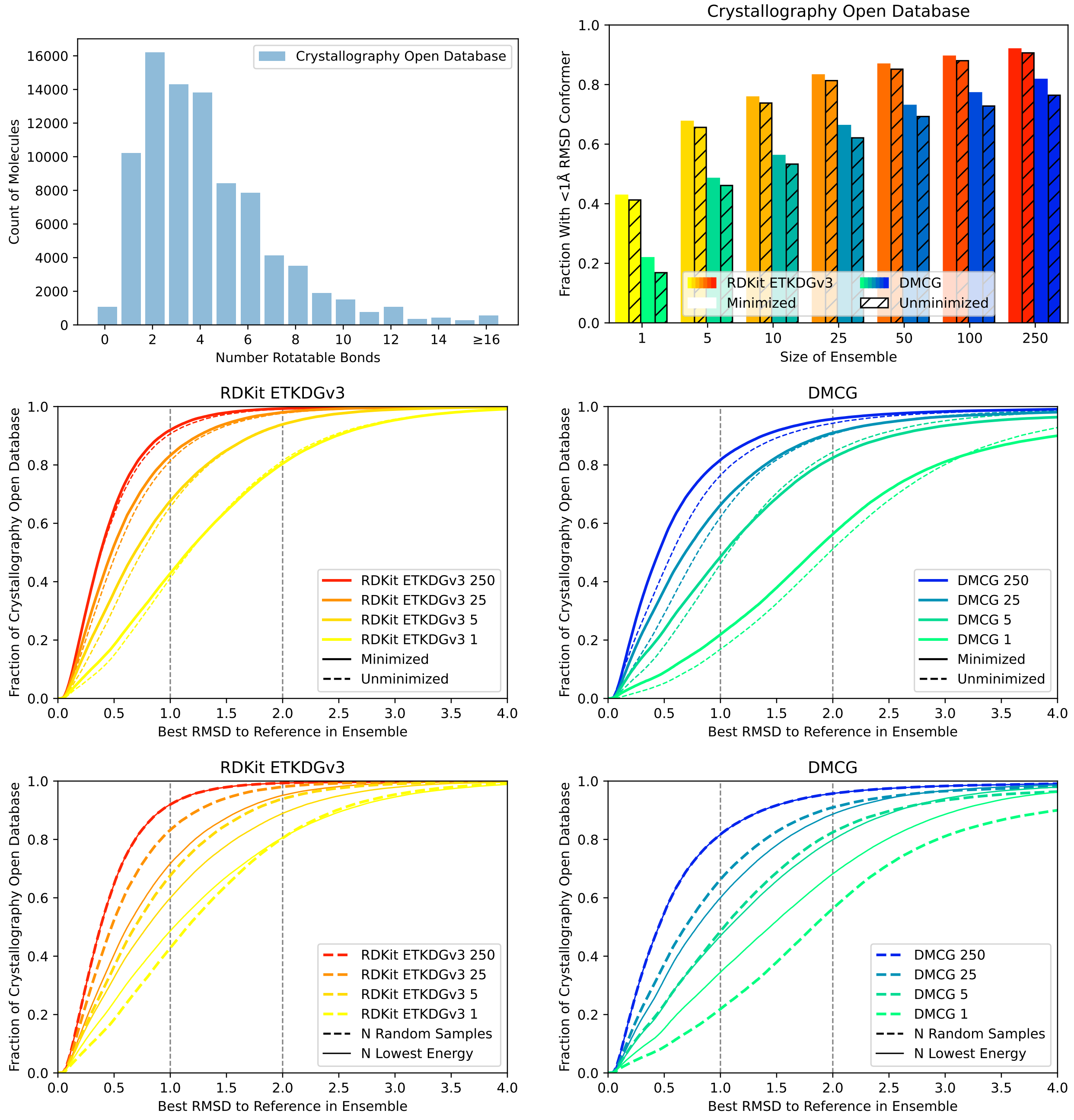

Figure S10: Evaluation of retrieval of conformations from the Crystallography Open Database.

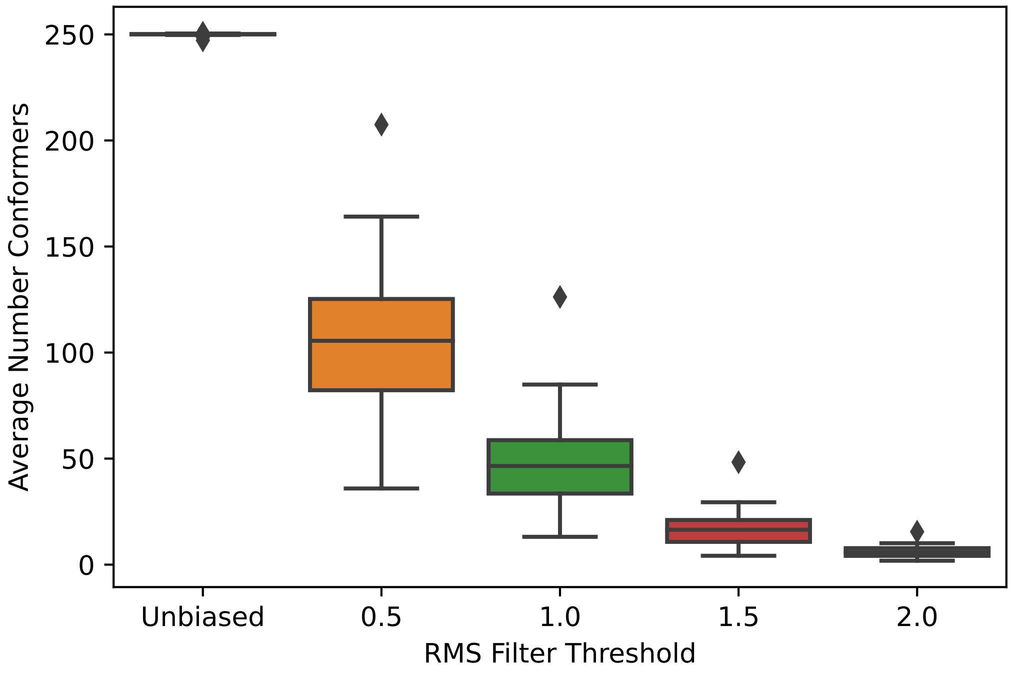

Figure S11: Distribution of the average number of conformers per a molecule for each DUDE target as the maximum number of allowed conformers varies.

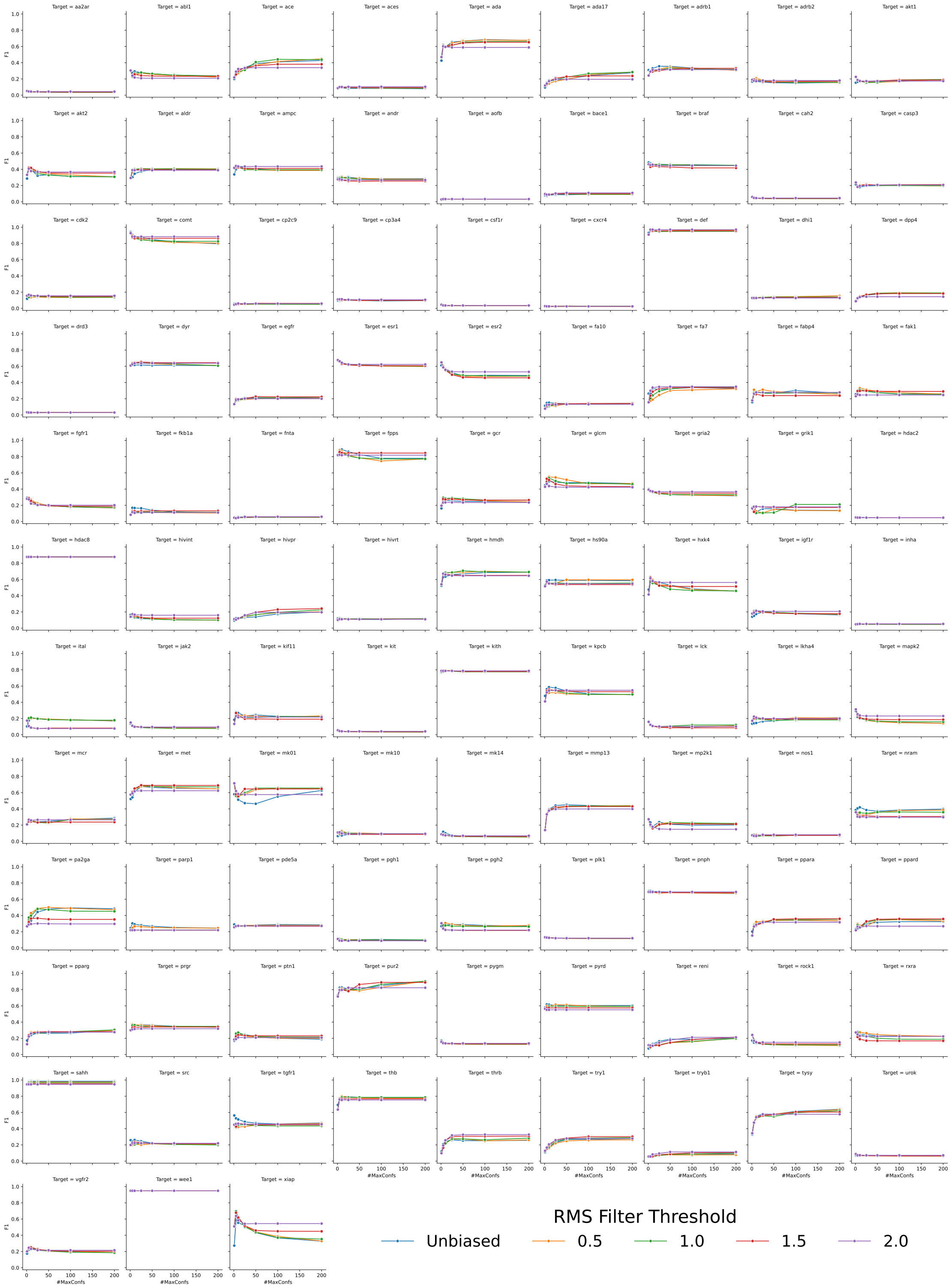

Figure S12: Relationship between best F1 score achieve through pharmacophore search and the maximum number of conformers allowed for each molecule for different RMSD filtering thresholds for all 102 DUDE targets.

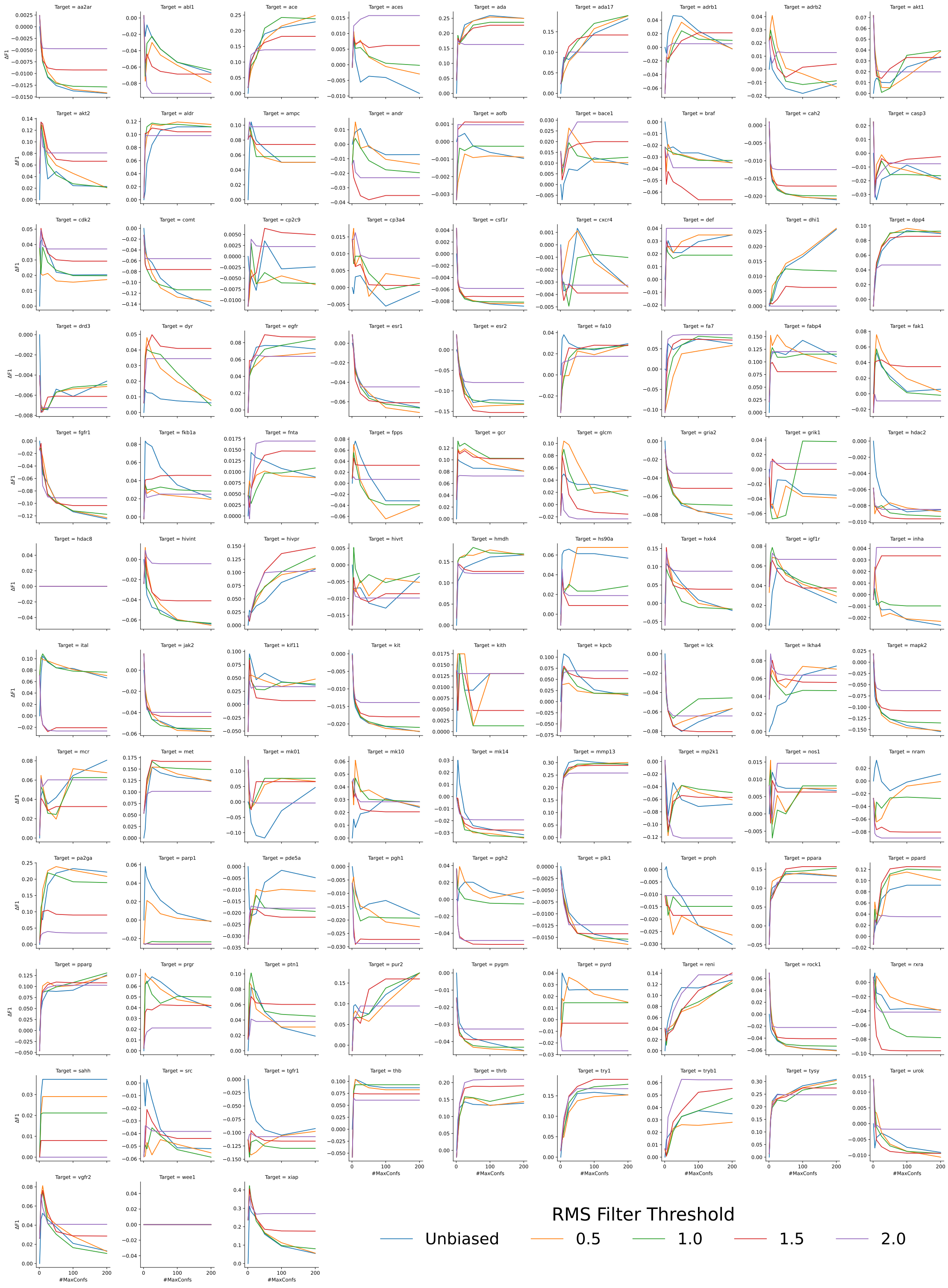

Figure S13: Relationship between ΔF1 score (difference with F1 using single conformer sampled without filtering or energy ranking) and the maximum number of conformers allowed for each molecule for different RMSD filtering thresholds for all 102 DUDE targets. Each subplot has its own y-axis scale to better illustrate trends.

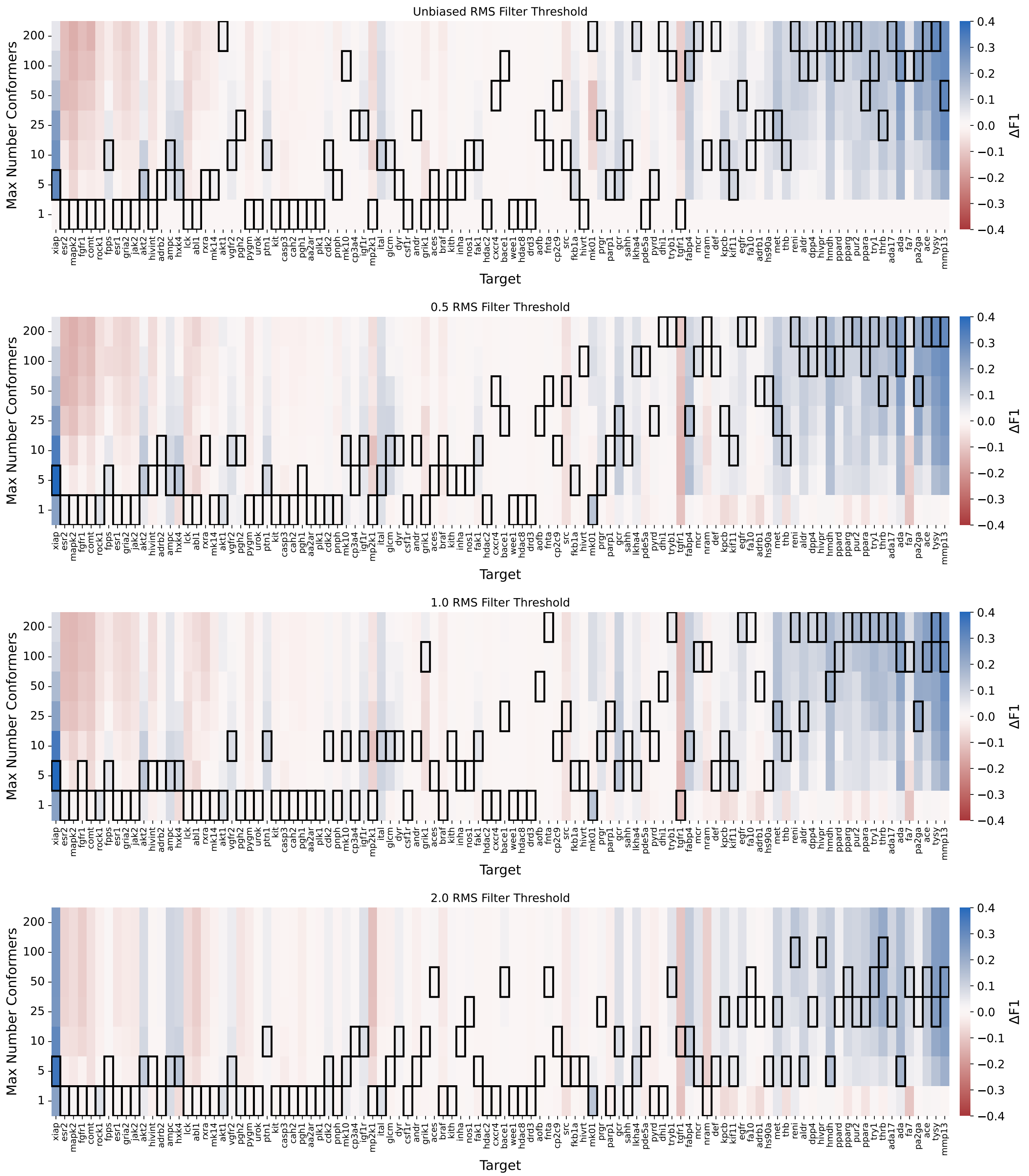

Figure S14: For each DUDE target, the difference in best achieved F1 score relative to the best F1 achieved by unbiased sampling of a single conformation is shown for different numbers of maximum allowed conformations. Conformers are selected by sorting by energy and then filtered by the specified RMS threshold. Targets are sorted by the slope of the best fit line through the conformer/F1 data for an RMS filter threshold of 0.5 (so that there is a consistent ordering on all figures). Box outlines highlight the choice of number of conformers that provides the highest F1 score.

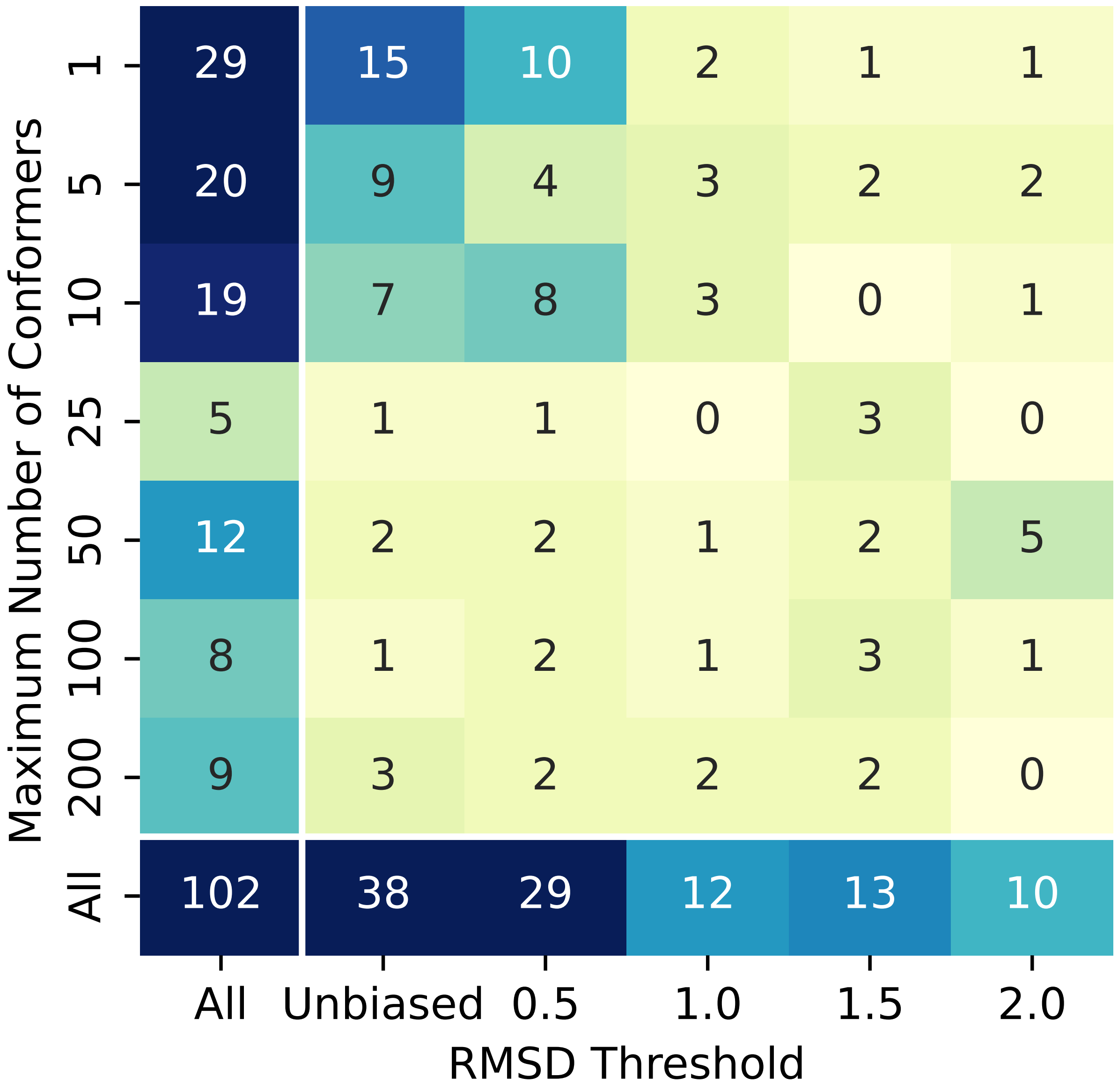

Figure S15: The number of targets that had their best F1 score for a given choice of maximum conformers and filtering threshold. Smaller, less filtered ensembles are preferred. If only a filtering threshold of 0.5 RMSD is considered, there are 29 targets with a best F1 using a single conformer, as reported in the main paper, but 19 of these have better F1 scores with a different filtering threshold.

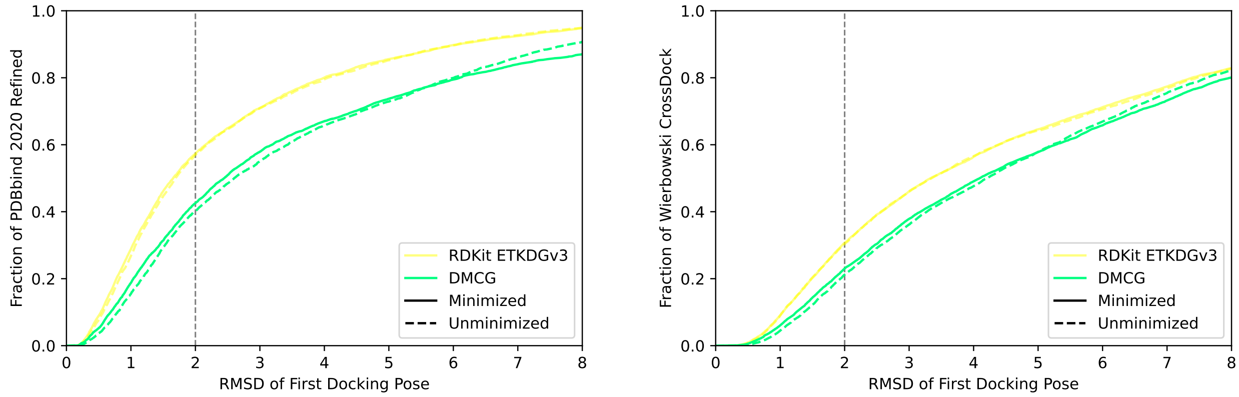

Figure S16: The effect of using an energy minimized pose from RDKit or DMCG as input for docking when redocking the PDBBind Refined set (left) and cross-docking (right). Minimization only minimally improves the results and is more important for DMCG.

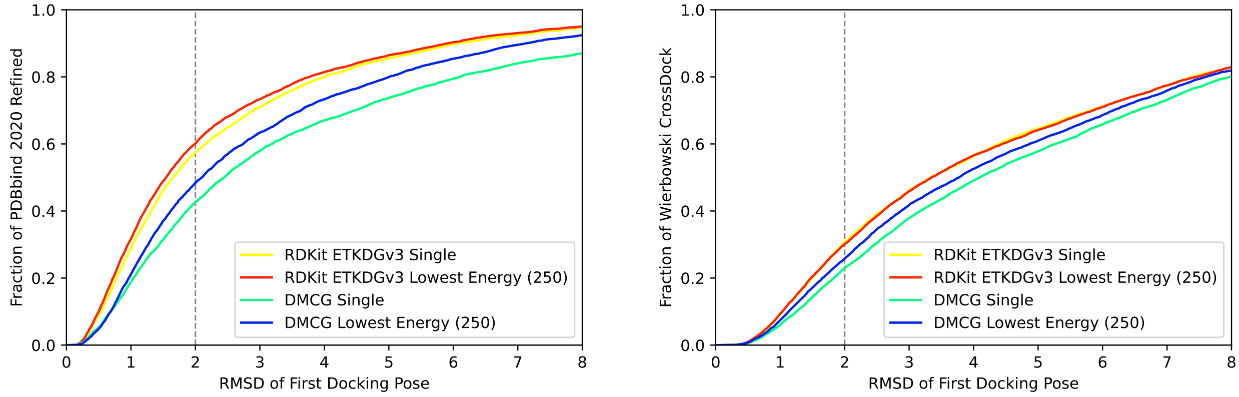

Figure S17: The effect of using the lowest energy pose (from an ensemble of 250) from RDKit or DMCG versus a randomly selected pose as input for docking when redocking the PDBBind Refined set (left) and cross-docking (right). RDKit significantly outperforms DMCG and benefits less from energy minimizing the generated poses.

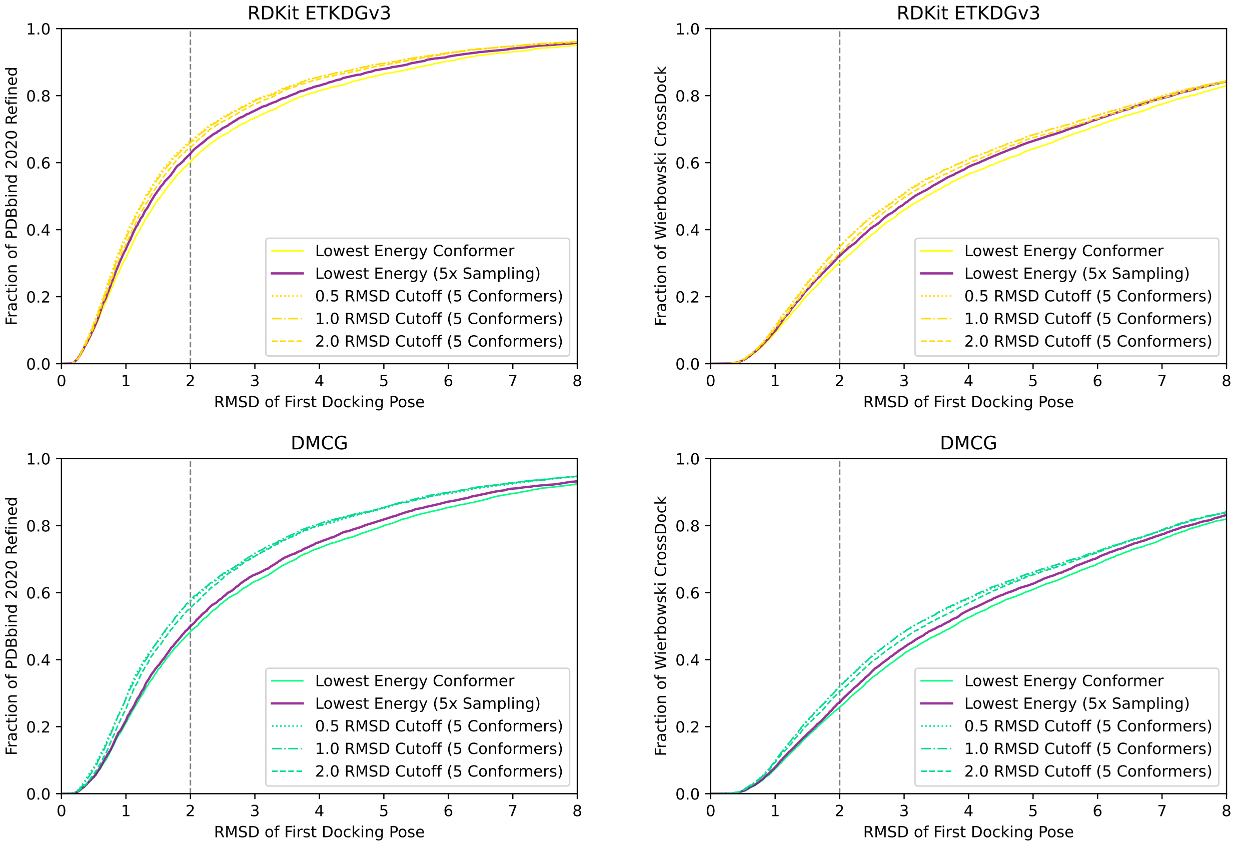

Figure S18: The effect of different criteria for constructing an ensemble five low energy conformers (from an ensemble of 250) from RDKit or DMCG when redocking the PDBBind Refined set (left) and cross-docking (right). As using five conformers increases the amount of sampling five fold, we also compare to docking a single lowest energy conformer with five times the amount of sampling (`--exhaustiveness 40`). Although docking samples the torsional space of ligands, the improvement observed from using an ensemble (largely independent of how the ensemble is selected) indicates an opportunity to improve docking results by also sampling non-torsional degrees of freedom.

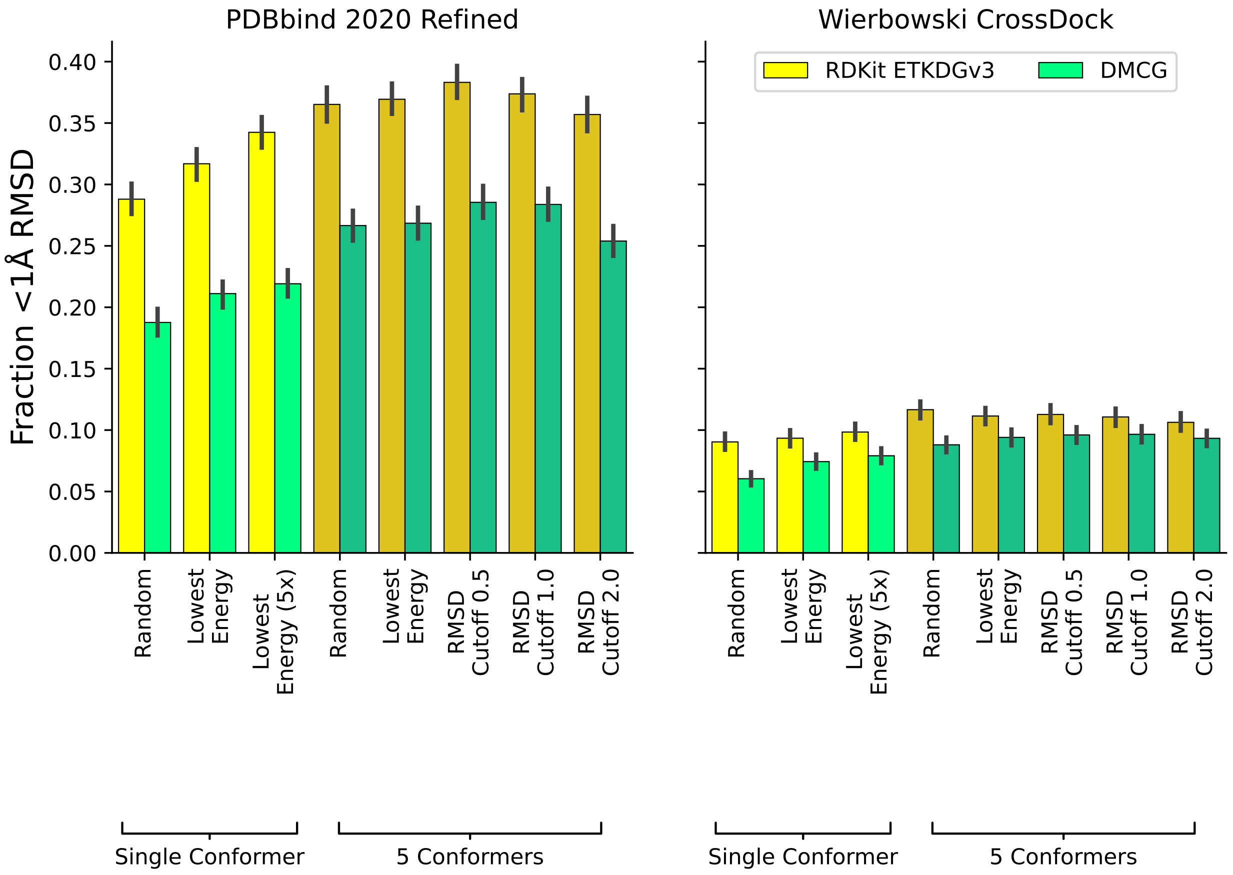

Figure S19: The effect of using different input conformer ensembles on docking performance as measured by the fraction of systems where a low (<1Å) RMSD pose is identified as the top ranked docked pose. Error bars indicate the 95% confidence interval determined from 1000 bootstraps.

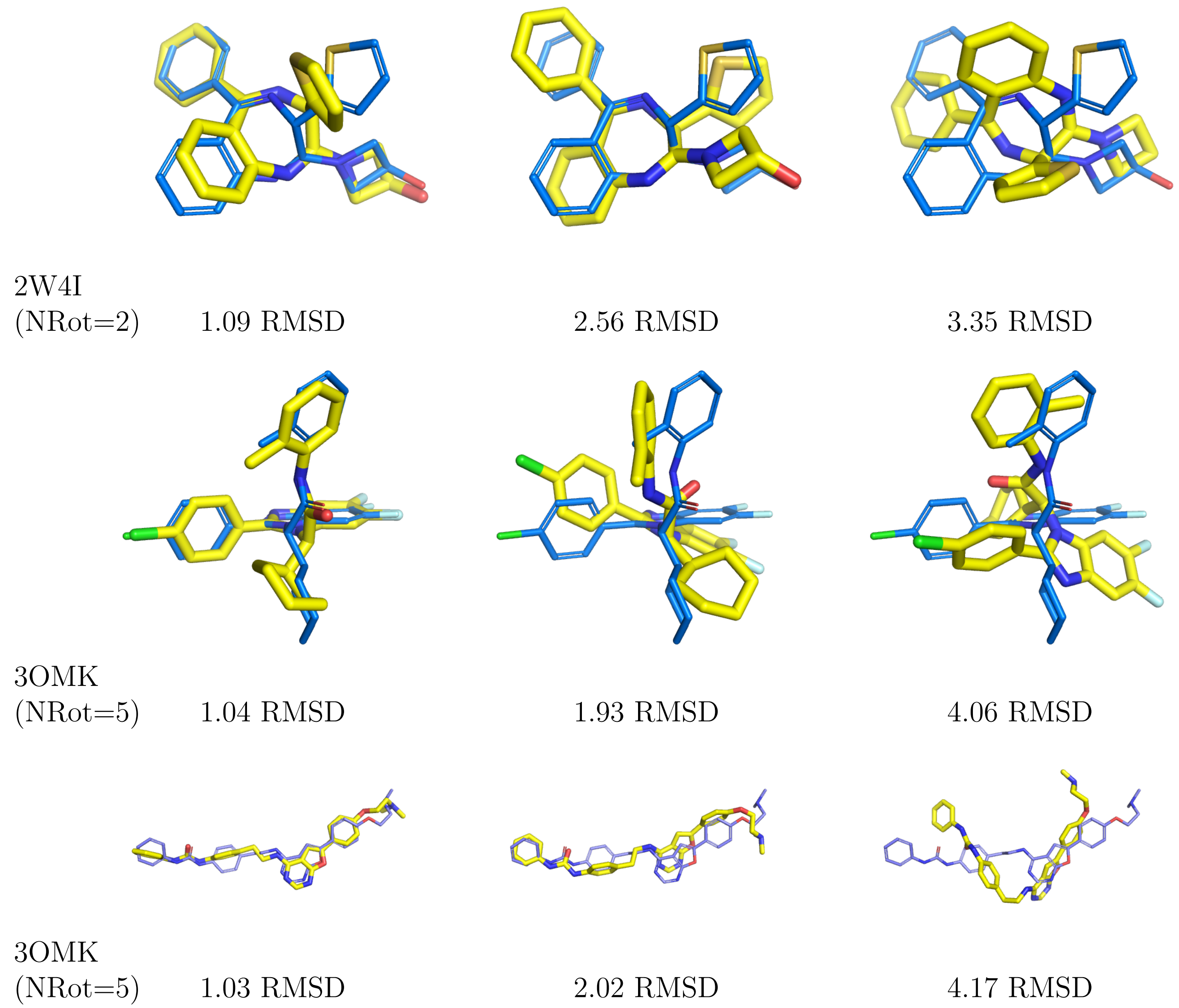

Figure S20: Examples of aligned conformations spanning different RMSDs for a selection of molecules with different numbers of rotatable bonds (NRot). The reference bioactive conformer is shown in think blue sticks and the generated conformer in yellow sticks.
